# What Do We Know About Contemporary Quality Improvement and Patient Safety Training Curricula in Health Workers? A Rapid Scoping Review

**DOI:** 10.3390/healthcare13121445

**Published:** 2025-06-16

**Authors:** Zoi Tsimtsiou, Ilias Pagkozidis, Anna Pappa, Christos Triantafyllou, Constantina Vasileiou, Marie Stridborg, Válter R. Fonseca, Joao Breda

**Affiliations:** 1 Department of Hygiene, Social-Preventive Medicine and Medical Statistics, School of Medicine, Aristotle University of Thessaloniki, 54124 Thessaloniki, Greece; pagoilias@gmail.com (I.P.); anna.pappa.246@gmail.com (A.P.); 2 WHO Athens Quality of Care and Patient Safety Office, World Health Organization Regional Office for Europe, 10675 Athens, Greece; triantafyllouc@who.int (C.T.); vasileiouc@who.int (C.V.); stridborgm@who.int (M.S.); fonsecav@who.int (V.R.F.); rodriguesdasilvabred@who.int (J.B.)

**Keywords:** quality of care, quality improvement, patient safety, health workers, training, curriculum, education, scoping review

## Abstract

Background and Objective: Despite growing emphasis on quality and safety in healthcare, there remains a limited understanding of how Quality Improvement and Patient Safety (QI/PS) training for health workers has evolved in response to global events like the COVID-19 pandemic and the WHO Global Patient Safety Action Plan. This rapid scoping review aimed to not only identify existing curricula but also uncover trends, innovation gaps, and global inequities in QI/PS education—providing timely insights for reshaping future training strategies. Methods: We searched MEDLINE and Scopus for English-language studies published between January 2020 and April 2024, describing QI and/or PS curricula across graduate, postgraduate, and continuing education levels. All healthcare worker groups were eligible, with no geographic limitations. Two reviewers conducted independent screening and data extraction; a third verified the results. Results: Among 3290 records, 74 curricula met inclusion criteria, with a majority originating from the US (58, 78.4%) and targeting physicians—especially residents and fellows (43/46, 93.5%). Only 27% of curricula were multidisciplinary. While traditional didactic (66.2%) and interactive (73%) approaches remained prevalent, curricula launched after 2020 introduced novel formats such as Massive Open Online Courses and gamification, with long-term programs uniformly leveraging web-based platforms. Common thematic content included Root Cause Analysis, Plan-Do-Study-Act cycles, QI tools, communication skills, and incident reporting. English-language peer-reviewed published literature indicated a marked lack of structured QI/PS training in Europe, Asia, and Africa. Conclusions: This review reveals both an uneven development and fragmentation in global QI/PS training efforts, alongside emerging opportunities catalyzed by digital transformation and pandemic-era innovation. The findings highlight a critical gap: while interest in QI/PS is growing, scalable, inclusive, and evidence-based curricula remain largely concentrated in a few high-income countries. By mapping these disparities and innovations, this review provides actionable direction for advancing more equitable and modern QI/PS education worldwide, whilst showcasing the need to systematically delve into QI/PS training in underrepresented regions.

## 1. Introduction

The need to “mind the gap” between standards and healthcare services with regard to quality of care (QoC) and patient safety (PS) has long been recognized. Τhe widespread interest in Quality Improvement (QI) and PS initiated in 1999 with the release of the United States (US) Institute of Medicine’s landmark report “To err is human” [1], while the publication of “Crossing the Quality Chasm” in 2001 led to the recognition of the necessity to include QI/PS efforts into medical practice and education [2]. To accelerate change, the 2030 Agenda for Sustainable Development, adopted by all United Nations Member States in 2015, introduced the 17 Sustainable Development Goals (SDGs), further emphasizing the need for QoC and PS [3]. Furthermore, recognizing that improving and ensuring PS is a growing challenge to health service delivery, the Global Patient Safety Action Plan 2021–2030 was adopted in 2021, aiming to provide strategic direction for all stakeholders for eliminating avoidable harm in healthcare and improving PS [4,5].

Improvement in QoC and PS as a priority in health sector policies and programs could be reinforced by training health workers, starting from their undergraduate education, and building further at the graduate, postgraduate, and continuous education levels. Noting the importance of healthcare professionals to meet this need, the World Health Organization (WHO) developed PS curriculum guides, initially in 2009, for medical schools [6], extended in 2011 to a multi-professional edition [7], while in 2012, a guide for developing training programs on PS research and improvement was released [8].

Previously published systematic and scoping reviews have identified studies reporting on QI/PS training curricula. Boonyasai et al. in 2007 published the first systematic review, identifying 39 curricula for clinicians (1980–2007), and demonstrating improvement in learners’ knowledge to perform QI [9]. Wong et al., in 2010, systematically reviewed published QI/PS curricula, identifying 27 targeting residents (2000–2009) and demonstrating that training can lead to meaningful improvements in clinical processes [10]. Expanding on Wong et al.’s work, Kirkman et al., in 2015, systematically reviewed 15 curricula for trainees/residents (2009–2014) [11]. In 2015, Starr et al. systematically reviewed studies describing QI curricula targeting healthcare professionals and their trainees/students, including 99 studies (2007–2013) [12]. Khurshid et al., in 2021, systematically reviewed 53 QI curricula for healthcare professionals (2009–2019), reporting that training healthcare professionals can have implications for improving healthcare systems [13]. In 2022, Li et al. explored in a scoping review the status of undergraduate and graduate nondegree QI/PS medical curricula in the US, identifying 38 in graduate medical education (2019–2022), most of which were specific to a particular specialty [14]. Ferro et al., in 2023, provided insights into the current state of the use of Quality and Safety Education for Nurses (QSEN) competencies in graduate nursing curricula in the US, identifying 10 relevant articles until 2021 [15]. Amaral et al., in 2023, through a scoping review, identified seven PS training programs for healthcare professionals (2010–2020) [16]. Finally, Agbar et al. 2023, through a systematic review and meta-analysis, identified 16 studies (2010 and 2020) that evaluated interventions to improve PS culture in inpatient care and general practice, outlining the importance of including all healthcare members involved in patient care and the need for occasional refresher training [17].

Notwithstanding the growing interest and focus on this field in healthcare settings, reflected by the increasing numbers of publications on QI/PS or solely PS curricula, evidence and understanding are limited on how QI/PS training for health workers has advanced and adapted in response to global turning points such as the increased digitization fueled by the COVID-19 pandemic and the adoption of the WHO Global Patient Safety Action Plan. Mapping the contemporary knowledge and unearthing trends, gaps, and inequities regarding training curricula and practices at a graduate, postgraduate, or continuous educational level on QI/PS in health workers are crucial in informing, developing, and implementing future innovative training interventions, ones that differ from previous training processes and modalities [18]. Delving into evidence-based training curricula is key in assuring sound, evidence-based policies and QI/PS training strategies [19]. In light of these considerations, a rapid scoping review was conducted to systematically outline what is known about the contemporary QI/PS curricula at the graduate, postgraduate, and continuous educational levels, inclusive of all health workers, since January 2020, without geographical restrictions. Bridging the gap between undergraduate training and clinical practice, this review aims to highlight the current international knowledge regarding QI/PS training curricula on the enhancement of health workers’ skills and competences to provide quality care, whilst taking advantage of the post-pandemic landscape, where learners are increasingly familiar with digital means.

## 2. Materials and Methods

A rapid scoping review design was chosen to address what is known about the contemporary curricula of training practices at the graduate, postgraduate, and continuous educational levels on QI/PS in health workers. This scoping review utilized the PCC (Population, Concept, Context) framework to precisely articulate the key components of the research question and inform the development of the search strategy, whilst defining the appropriate eligibility criteria [20,21,22].

Population: Health workers, classified according to the WHO international classification, including all five broad groupings: health professionals, health associate professionals, personal care workers in health services, health management and support personnel, and other health service providers not elsewhere classified [23].

Concept: Training curriculum on QI and/or PS, in terms of structure, teaching methods and content at the graduate, post-graduate, or continuous educational level.

Context: Any healthcare setting, without geographical restrictions.

The review protocol is registered on the Open Science Framework (https://doi.org/10.17605/OSF.IO/WV78J). PRISMA Extension for Scoping Reviews (PRISMA-ScR) guided the development of this report [21], as well as the WHO’s practical guide on rapid reviews [24].

### 2.1. Eligibility Criteria

Peer-reviewed journal articles were included if they were published between 1 January 2020 and 21 April 2024, described a training curriculum on QI/PS at the graduate, postgraduate, or continuous educational level in health workers, had an abstract, and were written in English. An article met the ‘QI/PS curriculum’ criteria if there was a description of any kind of organized learning of QI/PS [25]. The curriculum was included either if it described a conceptual approach to QI/PS principles, or it focused on specific dimensions of QoC, like equity and patient/person-centeredness, or on specific topics of PS (e.g., handoffs, non-technical skills/perioperative patient safety, etc.). Articles were excluded if they (a) did not describe the educational curriculum; (b) described educational interventions with a primary purpose other than QI/PS education; (c) referred only to undergraduate education; (d) were editorials, commentaries, or reviews; (e) the full text was not written in English; and (f) the full text could not be retrieved.

### 2.2. Data Sources and Search Strategy

A systematic search of PubMed and Scopus was conducted on 21 April 2024 to combine terms and keywords associated with the research question. The search strategy was drafted by an experienced academic librarian and further refined through team discussion. It generated a combination of MeSH terms of the main concepts in question and relevant free text words in the title/abstract to maximize the sensitivity of the search (online Supplemental File S1).

The data sources search returned 3290 results (PUBMED: *n* = 1447, SCOPUS: *n* = 1843) that were imported into Rayyan software [26].

### 2.3. Selection of Sources of Evidence

Using Rayyan software, the 1070 duplicates were deleted, leaving 2220 citations eligible for screening. Two reviewers independently and blindly conducted title/abstract screening. A calibration exercise with a random sample of 10 abstracts, conducted by both reviewers, was used to refine the inclusion and exclusion criteria. Articles were categorized as ‘include’, ‘exclude’, and ‘maybe’. The results were then unblinded, and screening discrepancies were discussed. In case of disagreement, articles were categorized as ‘include’. Two reviewers then shared the 197 full-text articles to be retrieved and assessed for eligibility, while a third reviewer verified the screening for accuracy. Disagreements on article selection and data extraction were resolved by consensus and discussion with other team members.

### 2.4. Data Charting Process and Data Items

Data from eligible articles were charted using an Excel data extraction tool designed for this study. The tool captured the relevant information on characteristics of the targeted learners and the setting where the training took place, while detailed information on the curriculum was extracted. It included the following fields: study publication information (author and year of publication, country), learner characteristics (profession, professional status, medical specialty), the name of the setting where it was delivered or the organization/institution that developed it, and curriculum characteristics (period of implementation, duration, program’s focus, program’s name, teaching methods, and educational content). Reviewers conducted a calibration exercise on three articles, which improved the data extraction sheet. Two reviewers extracted data after sharing the eligible articles, while the third reviewer verified the process for all the included articles for accuracy. Extraction difficulties or discrepancies were resolved by team discussion.

### 2.5. Synthesis of Results

The evidence quantitatively summarizes key characteristics of the targeted learners, the country of origin, and curricular description in terms of content focus area, teaching methods, duration, and structure. The extracted data are also presented in online Supplemental Files S2–S4, organized in tables.

## 3. Results

### 3.1. Selection of Sources of Evidence

**Figure 1:**
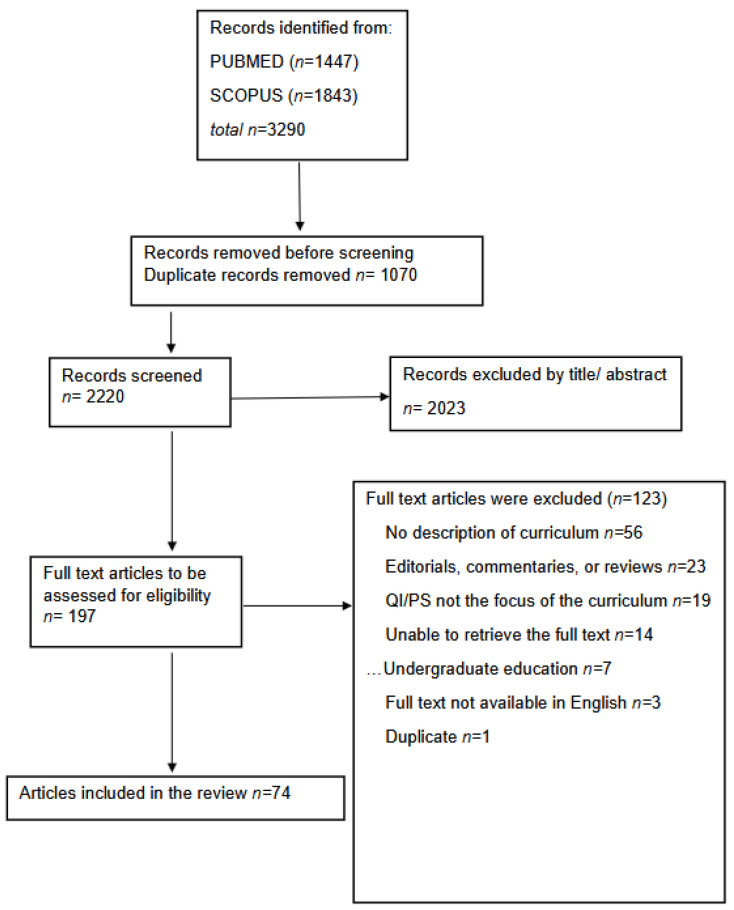
PRISMA-ScR flow diagram.

After duplicates were removed, based on the title and the abstract, 2023 were excluded, with 197 full-text articles to be retrieved and assessed for eligibility. Of these, 123 were excluded for the following reasons: 56 had no description of curriculum, 23 were editorials, commentaries, or reviews, 7 referred only to undergraduate training, 19 had other focus than QI/PS, 3 had no full text available in English, and 1 was duplicate—presenting the same intervention/setting in an article with a different title. We excluded 14 articles because we were unable to retrieve them. The remaining 74 articles were considered eligible for this review. The procedure is presented in the PRISMA ScR flow diagram (Figure 1).

### 3.2. Characteristics of Included Curricula

An overview of the characteristics of the targeted learners, the country of origin, and curricular description in identified curricula is illustrated in Table 1.

#### 3.2.1. Country

Most articles (58, 78.4%) reported US training programs. Of the remaining, six (8.1%) came from European countries (Ireland, The Netherlands, UK, Italy, and Norway), three (4.1%) from Canada, three (4.1%) from Australia, and two (2.7%) from Asian countries (Japan and Republic of Korea). Finally, one (1.4%) was developed as an international learning collaborative addressed to a mixed international audience, and one (1.4%) was developed by the American Society and delivered in different countries.

#### 3.2.2. Learners

With regard to the WHO classification of healthcare workers, all identified curricula addressed to healthcare professionals, 9.5% (*n* = 7) to health associate professionals, while 12 (16.2%) addressed health management and support personnel.

Most of the included curricula targeted physicians (*n* = 46, 62.2%), especially residents/trainees/fellows (in total *n* = 43/46, 93.5%, 38 residents/trainees, 4 fellows, and 1 both). Among the latter 43 QI/PS programs, 28 (65.1%) addressed to learners from one specific medical specialty [Internal Medicine (*n* = 9), Pediatrics (*n* = 3), Family Medicine (*n* = 3), Psychiatry (*n* = 2), General surgery (*n* = 2), Otorhinolaryngology (*n* = 2), Hematology/Oncology (*n* = 1), Maternal fetal medicine (*n* = 1), Neurology (*n* = 1), Physical Medicine and Rehabilitation (*n* = 1), Radiology (*n* = 1), Urology (*n* = 1), Cardiothoracic surgery (*n* = 1), while the rest to learners from several medical specialties. Among the 74 identified curricula, 3 (4.1%) targeted nurses, 2 (2.7%) pharmacists, and 1 (1.4%) physiotherapists. The remaining 20 (27%) interventions addressed more than one discipline. Four articles (5.4%) described curricula for postgraduate students, while three (4.1%) also addressed undergraduate students [31,36,99].

### 3.3. Curricular Description

#### 3.3.1. Content Focus Area

Among the included curricula, 27 (36.5%) were specified as addressing QI (described in detail narratively in online Supplemental File S2), 27 (36.5%) PS (online Supplemental File S3), and 20 (27%) both QI/PS (online Supplemental File S4). Among the 27 QI curricula, 5 (18.5%) focused on the improvement in specific QoC dimensions, and specifically equity (*n* = 2), patient/person-centeredness (*n* = 2), and efficiency (*n* = 1), while the majority (*n* = 22; 81.5%) used a broader QI conceptual approach. Furthermore, among the 27 PS curricula, 13 (48.1%) focused on specific topics, as handoffs/handovers (*n* = 4), event reporting (*n* = 3), Root Cause Analysis (*n* = 1), non-technical skills/communication/teamwork (*n* = 2), perioperative patient safety (*n* = 1), antibiotic stewardship (*n* = 1), and health worker’s well-being after PS events (*n* = 1), while most programs (14; 51.9%) used a broader PS conceptual approach.

#### 3.3.2. Teaching Methods

Most curricula employed a mixture of teaching methods, combining didactic and experiential learning. Didactic lectures/sessions were mentioned in 49 (66.2%) curricula, while in 33 (44.6%), online modules/educational material were used for self-study, either combined with didactic sessions or substituting them. Interactive sessions, including group discussions and case-based learning, were very commonly used either in face-to-face (*n* = 43, 58.1%) or distance learning (*n* = 6, 8.1%) or combining both modes (*n* = 5, 6.8%). The use of simulation was mentioned in 22 (29.7%) curricula. Project work was used in 37 (50%) curricula, either individually or in small groups, and its oral presentation was usually required. Participation in Morbidity and Mortality (M & M) conferences was included in six (8.1%) programs. Among the 11 training interventions launched from 2020 onwards [33,34,40,47,63,77,89,90,92,95,97], innovative teaching approaches like Massive Open Online Courses (MOOCs) (2 programs, 18.2%) and gamification (1 program, 9.1%) were also described. It is worth noting that among the latest 11 training curricula, 6 (54.5%) were conducted exclusively through web-based platforms [33,34,77,89,92,95], 2 (18.2%) were hybrid, combining online with in-person sessions [40,63], whilst the 3 (27.3%) face-to-face interventions were delivered only in programs with a single session and duration up to 3 h [47,90,97]. Virtual and hybrid training, simulations, MOOCs, and gamification constituted innovative teaching modalities that significantly shifted from traditional didactic approaches and were utilized in identified contemporary curricula.

#### 3.3.3. Educational Content

The curricula addressed a wide range of QI and PS content and were significantly heterogeneous (online Supplemental Files S2–S4). The most common contents were as follows: Root Cause Analysis (*n* = 29, 39.2%), PDSA cycle (*n* = 26, 35.1%), essential QI tools (*n* = 37, 50%), and communication/teamwork/non-technical skill education (*n* = 24, 32.4%). Human factor engineering, system thinking, as well as event reporting, and error disclosure were also covered in some programs (*n* = 6, *n* = 8m and *n* = 17, respectively), while introductory sessions to PS and the basic concepts of QI were included in fewer programs (*n* = 6 and *n* = 12, respectively). Already available and established online modules were also incorporated into 16 (21.6%) curricula, as modules from the Institute of Healthcare Improvement (IHI) Open School (*n* = 11, 14.9%), which was also used in training the trainers in two programs [36,64]. The online American Academy of Family Physicians (AAFP) TIPS QI modules were also included in one program.

#### 3.3.4. Duration and Structure of Training Curricula

The curricula varied widely in their duration and structure, ranging from a 1 h didactic lecture or interactive workshop to a 3-year curriculum across the residency. Among them, 28 (37.8%) had a duration of up to 3 h, 11 of them described a unique session, while 23 (31.1%) were delivered within a period of at least one year. Most curricula, 53 (71.6%), involved a combination of multiple sessions/activities

## 4. Discussion

We identified 74 articles describing training curricula at a graduate, postgraduate, or continuous educational level on QI/PS in health workers published between January 2020 and April 2024. Based on previous findings, our novel review seeks to shed light on the contemporary knowledge of the respective curricula addressed to all health workers, in light of the adoption of the WHO Global Patient Safety Action Plan and the acceleration of digitization instigated by the COVID-19 pandemic. Our findings indicate not only the increased interest in the development and delivery of QI/PS training interventions but also the remaining heterogeneity in their geographical origin, as well as in their content, design, structure, method of delivery, and duration of identified curricula.

### 4.1. Country and Learners: Comparison with Literature

Our findings underscore that despite the expanding interest in the development and delivery of QI/PS curricula, English-language, peer-reviewed publications outlining evidence-based, inclusive, and scalable training and interventions for health workers are still concentrated in a few high-income countries. Most of the identified QI/PS curricula were implemented in the US and targeted physicians, primarily residents, in accordance with former reviews [10,11,12]. The dominance of the US may reflect the incorporation of QI/PS in national curricula and recommendations by medical colleges [101]. Assimilating essential QI/PS competencies, the Accreditation Council for Graduate Medical Education (ACGME) promotes a culture of quality and safety across residency training [102], while according to the American Board of Medical Specialties (ABMS), QI competencies are required for initial acquisition and maintenance of board certification [103,104]. Likewise, in Canada, the CanMEDS framework for competent physicians [105], and in Australia, the National Safety and Quality Health Service Standards reflect continuous efforts to assure the expected quality and safety standards in provided care [106,107,108]. As the ACGME and CanMEDS mandate the active involvement of physicians in QI/PS activities, the number of studies reporting on the curricular design, content and evaluation in the US, followed by Canada and Australia, especially those throughout residency training, is expected to augment, emphasizing the importance of the development of national curricula and recommendations for health workers.

Among the identified curricula, two thirds addressed residents of one specific medical specialty, primarily Internal Medicine and Family Medicine, in line with Wong et al. [10], while increased interest was noted in Pediatrics. The rest addressed residents/fellows of several specialties, reflecting encouraging efforts to include QI/PS curricula in 13 medical specialties. As studies reporting on QI/PS curricula were scant amongst trainees and residents in surgical specialties, it is high time we upscaled training in these ‘high risk’ procedural and surgical residency programs. Two fifths of identified curricula targeted either other health workers or had a multidisciplinary audience. This could be attributed to the interest and acknowledgement of the importance of QI/PS from respective professional associations, like the American Association of Colleges of Nursing (AACN) [109]. Steering focus towards health workers and multidisciplinary teams reflects the need to develop shared understanding, processes, and vision for QI/PS in provided care among health workers, enhancing cooperation and promoting the development and improvement of a ‘safety culture’ in health settings [17].

### 4.2. Teaching Methods Comparison with Literature

Building on previous knowledge of well-established adult learning techniques identified as key factors for success in QI/PS training [9,10], experiential learning characterized most of the identified curricula. Yet, traditional methods, such as didactic sessions and lectures, were still an integral component in more than half of the reviewed curricula. In line with Kirkman et al. [11], our findings indicated the emerging popularity and utilization of interactive learning techniques such as group discussions and case- and project-based learning. Increased use of such modalities mirrors the value of debriefing, reflection, and learning from near-misses and adverse events, as well as widespread acceptance among residents [110,111,112]. In contrast with the infrequent use of web-based tools in Wong et al.’s review [10], the integration of online, asynchronous, self-paced modules, and learning material complements off-line training in contemporary curricula. However, online materials in the latter did not solely support didactic lectures, as some curricula relied solely on online self-studying [71,89,95,98]. The transition from offline to digital training methods and facilitation of distance learning may be attributed to the needs-based assessment of participants in respective studies as well as the accelerated digitization induced by the COVID-19 pandemic. All identified post-2020 training interventions with a duration of over three hours had incorporated blended or solely distance learning. Likewise, experiential learning in group or individual QI/PS projects is incorporated in half of the included curricula, allowing for the identification of ‘hazards’ in participants’ work environment and the application of acquired knowledge, skills, and QI methodology, in line with previous reviews [11,12].

Finally, our review ultimately highlights the contemporary methods of delivering QI/PS training to health workers, such as the use of MOOCs and gamification. Regarding the latter, and though excluded from our study due to the absence of an abstract, Congdon et al.’s Patient Safety Medical Escape Room constitutes a novel approach to PS training [113]. Early reports of the use of gamification in QI/PS curricula emerged in 2019 [114,115]. The use of novel training methods may increase participants’ excitement and engagement as well as spark interest in the training content [17,114].

### 4.3. Educational Content Comparison with Literature

Heterogeneity was identified in the QI/PS topics that health workers were trained on in the included curricula. The most common topics were Root Cause Analysis (RCA), Plan-Do-Study-Act (PDSA) cycle, and QI essential tools, with a high reference to the Fishbone diagram. An increasing focus on nontechnical skills, human factors, and systems thinking was noted among the studied curricula. RCA, general PS elements, error reporting, and systems thinking were also primarily incorporated in 2000–2009 curricula [10], whereas 2009–2021 curricula additionally focused on QI, communication, teamwork, human factors engineering, and nontechnical skills [11,17]. The ongoing focus on the latter, including communication, “Team Strategies & Tools to Enhance Performance & Patient Safety” (TeamSTEPPS) and “Situation, Background, Assessment, and Recommendation” (SBAR) techniques, also formerly noted [11,17], underscores their continued relevance as essential QI components in contemporary curricula. Specific PS topics such as handoffs/handovers and error reporting and disclosure are still the primary focus of many curricula, in line with a former review [17]. Our analysis identified a few programs providing solely a general and introductory overview of PS or QI, a finding that may reflect the maturity of some healthcare settings that delve into specific QI/PS elements, whereas offering introductory courses to healthcare workers in others is essential [17]. Compared to the suggested curriculum topics in the 2011 multiprofessional edition of the WHO PS curriculum guide [7], engaging with patients and carers, infection prevention and control, PS in invasive procedures, and medication safety were the least represented. Whilst the content depends on the duration of delivered curricula and the special needs of each context, future PS curricula could consider incorporating and training health workers on a wider breadth of PS topics from established, comprehensive guidelines with increased focus on empowering patients and carers to become more active members of their healthcare team and relevant specific PS topics. Moreover, some contemporary curricula use readily available, online, and established material, developed mainly by IHI [116], to train both health workers and trainers on QI/PS. Though noted in a former review [14], incorporating established modules in training curricula has increased in contemporary studies. Kazana et al. provided a comprehensive analysis of web-based, self-study QI resources for nursing professionals offered across eight websites [117]. Our findings suggest that the available online courses from various acknowledged sources could be tested in future curricula on QI/PS essential methodology.

Our findings unearth considerable variation in the duration and structure of identified QI/PS educational programs, in line with Wong et al. [10], spanning from single sessions of didactic lectures or workshops to curricula spanning across multiple years. It is worth noting that the majority of the curricula included consisted of multiple meetings, sessions, and activities across time. As each program sets specific goals and objectives, variability and absence of uniform QI/PS curricula design may explain the observed heterogeneity [17]. Standardized structures and guidelines on designing, delivering, and evaluating QI/PS curricula could allow the comparison and evaluation of outcomes in future interventions.

### 4.4. Limitations

Our rapid scoping review has some limitations. To begin with, we may have missed some relevant articles due to our search strategy, although no review can truly claim to find all relevant studies. To make our rapid review more feasible, as it served to inform and support European Region countries to pursue evidence-based training on QI/PS targeting all health workers, we were only able to systematically search PubMed and Scopus; hence, relevant studies may have been overlooked due to indexing. Yet, our abstracting strategy is in accordance with PRISMA-ScR guidelines and the WHO practical guide on rapid reviews mandating the use of at least two electronic databases [21,24]. We only included published studies in the English language, leading to the underrepresentation of non-English QI/PS curricula from ‘non-Western’ regions in our findings. Moreover, articles outlining curricula that were not available through the Hellenic Academic Libraries Link (HEAL-link) were not included in our synthesis, and no contact with the authors of non-accessible articles was pursued according to the WHO rapid review guide followed [24]. In efforts to base our rapid review on sound scientific evidence to assure evidence-informed decision making, QI/PS training, and policies [19], we pursued the inclusion of only peer-reviewed curricula in our findings and did not include grey literature. Even though we conducted an extensive database search, some relevant articles may have been unintentionally excluded from this review due to improper indexing or other factors. In this context, whilst language, publication, and access bias limited the curricula available for review and may have deterred us from comprehensively uncovering gaps and inequities in QI/PS training, our review managed to capture global efforts, inclusive of all health workers. Further adding to this issue is the significant heterogeneity across the included studies in terms of number and type of targeted participants, the course’s educational content, the employed teaching methods and modalities, which may have resulted in misclassifications in the quantitative synthesis of the results. The team sought to overcome this by having a double review at every stage of data abstraction.

## 5. Conclusions

This rapid scoping review identifies the target population, teaching modalities, educational content, and structure of contemporary QI/PS curricula for health workers at the graduate, post-graduate, and continuous educational levels, underscoring trends, emerging opportunities, and current disparities. In the past few years, an increasing trend in the development of PS educational interventions has been observed. A growing interest in designing and delivering QI/PS curricula targeted at non-physician health workers as well as to multi-disciplinary audiences is noted. Contemporary curricula innovatively approach delivery methods, combining traditional teaching methods with creative modalities such as MOOCs and gamification. Fueled by pandemic-era innovation and predominantly integrated in post-2020 studied curricula, the value of distance learning and web-based tools in achieving curricula objectives and participant engagement remains to be explored. The curricular incorporation of readily available and accessible QI/PS online courses, already developed and established by healthcare organizations and professional bodies, is also underscored. Formal integration of a QI/PS curriculum into graduate, post-graduate, and continuing education of health workers remains a work in progress internationally, especially in most European, Asian, and African countries, where the need for national training curricula and recommendations promoting a culture of quality and PS is emerging. Moving forward, as the majority of evidence-based interventions are predominantly developed and run in high-income nations, mapping disparities and innovations is key in advancing contemporary, yet equitable QI/PS education.

## Figures and Tables

**Table 1:** Overview of the identified curricula characteristics (*n* = 74).

Characteristics of Included Curricula		*N* (%)	Identified Studies
Country of origin	US Canada Australia UK Ireland Netherlands Italy Norway Japan Republic of Korea International learning collaborative US-developed with international target audience	58 (78.4%) 3 (4.1%) 3 (4.1%) 2 (2.7%) 1 (1.4%) 1 (1.4%) 1 (1.4%) 1 (1.4%) 1 (1.4%) 1 (1.4%) 1 (1.4%) 1 (1.4%)	[27,28,29,30,31,32,33,34,35,36,37,38,39,40,41,42,43,44,45,46,47,48,49,50,51,52,53,54,55,56,57,58,59,60,61,62,63,64,65,66,67,68,69,70,71,72,73,74,75,76,77,78,79,80,81,82,83,84] [85,86,87] [88,89,90] [91,92] [93] [94] [95] [96] [97] [98] [99] [100]
Learners	Health professionals Health management and support personnel Health associate professionals	74 (100%) 12 (16.2%) 7 (9.5%)	[27,28,29,30,31,32,33,34,35,36,37,38,39,40,41,42,43,44,45,46,47,48,49,50,51,52,53,54,55,56,57,58,59,60,61,62,63,64,65,66,67,68,69,70,71,72,73,74,75,76,77,78,79,80,81,82,83,84,85,86,87,88,89,90,91,92,93,94,95,96,97,98,99,100] [31,36,60,61,72,75,77,79,84,89,95,96] [31,39,41,67,75,76,79]
	Physicians Residents/trainees/fellows Several medical specialties Internal Medicine Pediatrics Family Medicine Psychiatry General surgery Otorhinolaryngology Hematology/Oncology Maternal fetal medicine Neurology Physical Medicine and Rehabilitation Radiology Urology Cardiothoracic surgery Multidisciplinary Postgraduate students Nurses Pharmacists Physiotherapists	46 (62.2%) 43 (58.1%) 10 (13.5%) 9 (12.2%) 3 (4.1%) 3 (4.1%) 2 (2.7%) 2 (2.7%) 2 (2.7%) 1 (1.4%) 1 (1.4%) 1 (1.4%) 1 (1.4%) 1 (1.4%) 1 (1.4%) 1 (1.4%) 20 (27%) 4 (5.4%) 3 (4.1%) 2 (2.7%) 1 (1.4%)	[27,28,29,32,33,35,37,38,40,42,43,44,46,47,48,49,50,51,52,53,54,56,57,58,61,62,63,65,66,68,70,71,73,74,77,78,80,81,82,83,85,86,87,93,97,100] [27,28,29,32,33,35,37,38,40,42,43,44,46,47,48,49,50,51,52,53,54,56,57,62,63,65,66,68,70,71,73,74,77,78,80,81,82,83,85,86,87,93,97] [27,28,29,33,37,38,51,57,62,78] [32,46,52,56,63,74,81,83,87] [44,68,80] [40,65,70] [47,86] [35,73] [66,85] [82] [77] [54] [42] [49] [43] [48] [30,31,34,36,41,55,59,60,64,67,72,75,76,79,84,85,89,92,94,95] [45,88,96,99] [69,88,98] [39,91] [90]
Curricular description	Content focus area Quality Improvement Conceptual approach Equity Patient/person-centeredness Efficiency Patient Safety Conceptual approach Handoffs Event reporting Non-technical skills Root Cause Analysis Perioperative patient safety Antibiotic stewardship Health workers’ wellbeing Quality Improvement and Patient Safety	27 (36.5%) 22 (29.7%) 2 (2.7%) 2 (2.7%) 1 (1.4%) 27 (36.5%) 14 (18.9%) 4 (5.4%) 3 (4.1%) 2 (2.7%) 1 (1.4%) 1 (1.4%) 1 (1.4%) 1 (1.4%) 20 (27%)	[27,30,31,34,35,36,42,44,46,54,61,62,64,65,69,70,73,78,83,86,87,89,90,91,92,96,100] [27,30,35,36,42,46,54,61,64,65,69,70,73,78,83,86,87,89,91,92,96,100] [34,44] [31,90] [62] [28,29,38,39,41,43,47,48,55,58,60,63,67,68,72,74,75,76,79,80,81,82,84,85,88,97,98] [29,38,39,43,47,55,60,63,72,80,81,82,88,98] [41,58,67,68] [28,79,97] [48,75] [84] [85] [76] [74] [32,33,37,40,45,49,50,51,52,53,56,57,59,66,71,77,93,94,95,99]
	Teaching methods		
	Interactive sessions		
	Group discussions	53 (71.6%)	[29,30,31,32,33,34,35,36,37,39,42,43,44,46,48,52,53,54,55,56,57,58,59,60,61,62,63,64,66,67,70,72,73,74,75,78,80,81,82,83,84,85,86,87,88,90,91,93,94,96,97,99,100]
	Case-based learning	29 (39.2%)	[28,29,31,32,33,37,38,40,43,44,47,51,54,57,58,63,72,74,76,79,81,82,83,84,85,86,98,99,100]
	Mode of delivery		
	Physical sessions	43 (58.1%)	[28,29,30,31,36,38,39,40,43,44,46,47,48,51,52,53,54,56,57,58,59,64,66,67,70,72,73,74,75,79,80,81,82,84,85,88,90,91,93,94,97,100]
	Virtual sessions	6 (8.1%)	[33,34,60,83,96,98]
	Hybrid sessions	5 (6.8%)	[50,55,63,78,86,99]
	Project work	37 (50%)	[27,29,32,33,34,35,36,37,40,42,43,46,49,50,51,54,55,56,57,59,61,62,63,65,69,70,73,77,78,83,86,87,92,93,94,99,100]
	Individual	9 (12.2%)	[40,42,50,61,77,86,92,93,94]
	Group	28 (37.8%)	[27,29,32,33,34,35,36,37,43,46,49,51,55,56,57,59,62,63,65,69,70,73,78,83,87,94,99,100]
	Didactic lectures	49 (66.2%)	[27,28,31,33,34,35,36,38,39,40,41,42,43,44,45,47,48,49,50,51,53,54,55,56,57,59,60,62,65,66,68,69,70,72,73,75,79,80,81,82,83,85,86,87,88,94,96,97,99]
	Online modules/educational material	33 (44.6%)	[27,29,30,33,36,40,41,42,44,45,48,49,52,53,54,55,56,58,59,60,61,63,66,69,72,73,76,77,78,91,96,98,99]
	Presentation of project work	27 (36.5%)	[27,29,32,33,34,35,36,40,42,43,46,49,50,54,55,56,59,61,62,63,65,73,77,78,83,87,100]
	Simulations	22 (29.7%)	[28,39,41,44,48,50,53,56,57,67,68,72,74,81,82,84,88,90,94,96,97,99]
	Morbidity and Mortality conferences	6 (8.1%)	[40,43,50,54,56,57]
	Massive Open Online Courses	2 (2.7%)	[89,92]
	Gamification	1 (1.4%)	[95]
	Educational Content Essential QI tools Root Cause Analysis PDSA Non-technical skills Event reporting and errors disclosure Established online modules IHI Open School AAFP TIPS QI Introduction to QI Systems thinking Introduction to PS Human factors engineering	37 (50%) 29 (39.2%) 26 (35.1%) 24 (32.4%) 17 (23%) 16 (21.6%) 11 (14.9%) 1 (1.4%) 12 (16.2%) 8 (10.8%) 6 (8.1%) 6 (8.1%)	[27,29,30,32,33,34,35,36,37,39,43,46,49,50,54,56,57,59,60,61,63,64,65,69,71,73,77,78,82,83,84,86,87,92,93,96] [28,29,30,32,34,38,39,40,43,47,49,51,53,56,57,59,60,63,70,71,72,77,78,81,82,83,84,87,98] [32,33,35,36,46,49,50,51,52,53,56,59,61,62,64,65,67,70,71,73,78,83,86,87,92,100] [30,36,41,44,48,49,50,53,55,57,58,65,67,69,71,74,75,77,79,85,88,89,99,100] [28,32,33,38,47,53,56,63,66,71,72,77,79,80,81,84,97] [25,34,38,41,43,47,52,53, 54,59,62,64,67,68,70,71] [27,36,45,49,54,55,56,61,64,66,73] [40] [30,36,40,62,66,70,71,77,78,83,96,100] [37,40,61,63,71,81,84,99] [52,63,66,71,72,81] [29,49,50,53,60,77]
	Duration Up to 3 h One year or more Structure Multiple sessions One session	28 (37.8%) 23 (31.1%) 53 (71.6%) 11 (14.9%)	[27,28,31,35,36,37,39,46,47,49,50,51,54,56,58,62,63,70,74,75,79,81,82,83,84,85,90,97] [28,30,38,40,47,48,49,52,53,54,58,59,63,67,71,74,75,78,82,85,88,91,92] [27,29,30,32,33,34,35,36,37,38,40,41,42,43,44,46,48,49,50,51,52,54,55,56,57,59,60,61,62,63,65,66,67,69,70,73,76,77,78,79,80,81,83,84,86,87,88,91,93,94,96,99,100] [28,31,39,47,58,74,75,82,85,90,97]

## Data Availability

All data are available online in Supplemental Files S2–S4.

## Supplementary Materials

The following supporting information can be downloaded at: https://www.mdpi.com/article/10.3390/healthcare13121445/s1. File S1: Full electronic search strategy for both databases used; File S2: Narrative presentation of the 27 training curricula described as addressing Quality Improvement (QI)—either with a conceptual approach to QI or focusing on specific dimensions of quality of care—for graduate, postgraduate or continuous education of health workers; File S3: Narrative presentation of the 27 curricula described as addressing Patient Safety (PS)-either with a conceptual approach or focusing on specific topics of PS-for graduate, postgraduate or continuous education of health workers; File S4: Narrative presentation of the 20 curricula described as addressing Quality Improvement and Patient Safety (QI/PS) for graduate, postgraduate or continuous education of health workers.
