# Supplementary material for: What Do We Know About Contemporary Quality Improvement and Patient Safety Training Curricula in Health Workers? A Rapid Scoping Review
Source: Healthcare (Basel). 2025 Jun 16;13(12):1445. doi: 10.3390/healthcare13121445 (PMC12193159; doi:10.3390/healthcare13121445)
Supplement: Supplementary file 1 [file healthcare-13-01445-s001.zip › File S1_Full electronic search strategy for both databases used.pdf]

Full electronic search strategy used for both databases

The final full search strategy for PubMed was:

(("curriculum"[MeSH Terms] OR "curricul*"[Title/Abstract] OR "education*"[Title/Abstract] OR "training"[Title/Abstract]) AND ("education, graduate"[MeSH Terms] OR "graduate"[Title/Abstract] OR "education, continuing"[MeSH Terms] OR "continuing"[Title/Abstract] OR "postgraduate"[Title/Abstract]) AND ("patient safety"[MeSH Terms] OR "patient safety"[Title/Abstract] OR "quality improvement"[MeSH Terms] OR "quality improvement"[Title/Abstract] OR "quality of care"[Title/Abstract] OR "quality of health care"[Title/Abstract] OR "quality of healthcare"[Title/Abstract])) AND (2020:2024[pdat]) AND ("english"[Language])),

while for Scopus:

TITLE-ABS-KEY (curricul* OR education* OR training) AND TITLE-ABS-KEY (graduate OR continuing OR postgraduate) AND TITLE-ABS-KEY ({patient safety} OR {quality improvement} OR {quality of care} OR {quality of health care} OR {quality of healthcare}) AND PUBYEAR > 2019 AND PUBYEAR < 2025 AND (LIMIT-TO (LANGUAGE , "English"))
