# Supplementary material for: What Do We Know About Contemporary Quality Improvement and Patient Safety Training Curricula in Health Workers? A Rapid Scoping Review
Source: Healthcare (Basel). 2025 Jun 16;13(12):1445. doi: 10.3390/healthcare13121445 (PMC12193159; doi:10.3390/healthcare13121445)
Supplement: Supplementary file 1 [file healthcare-13-01445-s001.zip › File S2_Narrative presentation of the 27 QI training curricula.pdf]

Narrative presentation of the 27 training curricula described as addressing Quality Improvement (QI) - either with a conceptual approach to QI or focusing on specific dimensions of quality of care - for graduate, postgraduate or continuous education of health workers.

**Source (First author, Year)**	**Country**	**Setting and/or developed by**	**Learners**	**Curriculum's/ program's title**	**Content focus area**	**Duration**	**Teaching methods/ Structure**	**Educational content***	**Period of implementation**
Ahuja, 2021 [27]	USA	Yale School of Medicine	Physicians-residents (GS, Plastic Surgery PGY2-3)	ns**	QI	9 months	1. Didactic lectures (2 hours, every other week for the first 3 months)<br>2. Project-based learning (group project)<br>3. Web-based learning (HI Open School modules)<br>4. Mentorship<br>5. Project presentation to mentor and peers	IHI Open School modules (QI 101–Q105, PS 101–105, TA 101, PFC 101, and L 101); History of QI; SMART aims; Key driver diagrams; Process mapping; Run charts; Variation; PS; Presentation of local quality improvement leaders' work with focus on interventions, reliability, and sustainability	2019-2020
Berger, 2020 [30]	USA	Illinois Surgical Quality Improvement Collaborative (ISQIC)	Physicians-(Surgical), hospital team members (surgical domain)	ISQIC Quality Improvement Curriculum	QI	1 year	1. Workshops (in-person, bi-annual meetings)<br>2. Web-based learning (30 min modules, *n*=10, webinars)<br>3. Coaching and mentorship	Project Management; Teamwork; Leading Change; QI Principles; Tools; RCA; FMEA; Toyota Six Sigma methodology (DMAIC); Example DMAIC Projects; DMAIC Skills Practice; Effectively Managing QI Projects; Using Data to Make the Case for Change; Utilizing FMEA to identify Local Opportunities; Introduction to ISQIC; Introduction to ACS NSQIP; How to build and lead effective QI teams; Accessing and Presenting Data	2015
Block, 2020 [31]	USA	Donald and Barbara Zucker School of Medicine at Hofstra/Northwell	Physicians-residents (IM), Medical, Physician assistant,	Improving Patient Access, Care, and Cost through Training	QI, Person-centeredness	3 hours	Workshop (Didactic lectures; Reflective activity; Group-based and Case-based learning)	Patient- Versus Provider-Centered Care; PCMH Principles; Continuity: unique challenges and definition in a team-based practice; Approaches to measuring access; Innovative approaches to improve access;	2017

			Pharmacy, Clinical psychology students, faculty, medical office assistant, practice coordinator	(IMPACcT) program					
Burra, 2022 [86]	Canada	University of Toronto	Physicians-residents (Psychiatry PGY 3)	ns**	QI	5 half-days	1. Seminars [in person or online seminars, Didactic sessions; Multimedia (video, excerpt from novel); Case-based learning; Group-based learning (i.e. think-pair-share); Games]<br>2. Project-based learning (individual project)	IHI Model for Improvement; Quality of care (dimensions); QI; SMART aim; PDSA; Process change; Data collection; Data visualization; Data analysis; QI tools (5 Whys, 5W2H, Fishbone diagram, Process map); Data for research vs Data for QI; Outcome/process/balancing measures; Run chart; Control chart; Pareto chart	2006-Updated from 2019- (in-person) 2020- ns (virtual)
Cheney-Peters, 2023 [34]	USA	Thomas Jefferson University	Physicians-trainees [Emergency Medicine (EM), Family Medicine (FM), General Surgery (GS), Internal Medicine (IM), Neurology,	EQUIP [ECHO (Extension for Community Healthcare Outcomes) Quality Improvement Program] for Equity	QI, Health equity	10 months	1. Group meetings (hub-and-spoke model, monthly sessions)<br>2. Didactic lectures<br>3. Team guides<br>4. Debriefing and feedback<br>5. Project-based learning (group project)<br>6. Project presentation at QI conference	Didactic lectures: Healthcare disparities; Identification of Community partners; Quality measures; QI intervention design; Community engagement; Data analysis<br>Team guide topics: Identification of Healthcare disparity/gap; Health equity focus on RCA; Causal chains in RCA; Specific, Measurable, Achievable, Relevant, and Time-Bound (SMART) aim; Identification of structure/outcome/process/balancing measures; Common interventions for healthcare disparities; Effort vs Impact matrix; Process map	2021 (hybrid)

			Neurosurgery, Obstetrics & Gynecology, Ophthalmology, Pathology, Physical Medicine and Rehabilitation (PM&R), Psychiatry, Radiology], population health graduates, faculty						
Collins, 2021 [87]	Canada	University of Alberta	Physicians-residents (IM PGY1-3) and faculty	Evidence-based Practice for Improving Quality (EPIQ) training course	QI	6 months	1. Workshops (4 hours)<br>2. Didactic lectures<br>3. Group-based learning (work groups)<br>4. Coaching<br>5. Project-based learning (participation in active QI projects)<br>6. Project presentation	Problem identification; RCA; PDSA; Intervention determination; Process mapping; Identifying measures; Completing an Aim and Change form	2017-2019
Colvin, 2021 [35]	USA	Cleveland Clinic Foundation	Physicians-trainees (Surgery all levels)	QIC (Quality Improvement Curriculum)	QI	11 weeks	1. Interactive workshops (1.5 hours, *n*=4)<br>2. Didactic lectures	Introduction to process improvement; Problem statement; Aim statement; Effective teams and roles; Stakeholders; Process mapping; PDSA; Performance measures; Describing the current state; Fishbone; 5 Whys; Decision matric;	2018

							3. Group-based learning, discussion and feedback<br>4. Project-based learning (group project, focused on PS,<br>5. Project presentation	Countermeasures; Priority payoff matrix; Change management: making the switch & implementing countermeasures; Effort vs Impact matrix; Data analysis	
Dulay, 2020 [36]	USA	San Francisco VA Health Care System's (SFVAHCS) Center of Excellence in Primary Care Education (CoEPCE) program	Physicians-trainees (IM PGY2), Nursing students (gerontolog y Primary Care Nurse Practitioner s) Pharmacists , Psychologis ts (postdoctor al fellows)	ns**	QI	9 months	1. Didactic lectures (1 hour)<br>2. Group-based learning and discussion<br>3. Project-based learning (group project)<br>4. Mentorship<br>5. Web-based learning (IHI Open School modules)<br>6. Debriefing<br>7. Project presentation (poster format, abstract and publication work)	Teambuilding; Interprofessional collaboration; IHI model for improvement; Lean model for transformation; Institute of Medicine's (IOM) characteristics of High Quality Care; Key QI agencies; IHI Open School modules; Introduction to QI; QI methodology; Commonly used QI tools; Performance Measures; Facility Quality Gaps; Effort/yield analysis; SMART aims; Microsystem/Gap Analysis; Process mapping; Flow chart; Fishbone diagrams; Cause and effect diagram; Gemba walk; Approach to System Change; Teamwork; PDSA	2011-2018
Dwyer, 2022 [89]	Australia	University of Tasmania	Nursing staff, Allied health practitioner s, Medical officers, Administrat ive staff	Healthcare Redesign MOOC	QI	6 weeks	MOOC (6 modules) [Multimedia (video, interactive quizzes, links for further reading); Case-based learning; Reflective exercises]	Healthcare redesign; Theories for redesigning Healthcare; Techniques for Engaging People; Challenges in redesign (Team motivation, Engagement of busy clinicians, Generating shared solutions, Negativity, Effective group facilitation); Communication and Engagement skills; Understanding and Addressing the Problem; Sustainability	2021 (launched)

Grover, 2021 [42]	USA	Washington University	Physicians-residents (PM&R PGY1-4)	PM&R Residency Quality Improvement and Research Curriculum	QI	1 academic year	1. Didactic lectures<br>2. Educational material (pen-and-paper and web-based resources)<br>3. Group discussions<br>4. Project-based learning (individual project)<br>5. Coaching on individual QI projects, publication and presentation activities<br>6. Project presentation (podium, posters)<br>7. Newsletter highlighting accomplishments (bi-monthly)<br>8. Literature review	QI; SMART; Outcome metrics; QI and research project methodology: Study design; Data analysis; Publication; Presentation basics; QI project rationale; design and outcome presentation	2015
Hernandez, 2022 [44]	USA	Johns Hopkins All Children's Hospital	Physicians-trainees (Pediatrics PG1-3)	Leaders in Health Equity (LHE)	QI, Health equity	PGY1: 1.5-2-day sessions (8 hours)<br>PGY2,3: 1-day session (8 hours)	1. Educational material (journal articles, policy statements, case descriptions, self-assessments)<br>2. Didactic lectures<br>3. Group discussions, reflection and debriefing<br>4. Individual introspection<br>5. Interactive exercises<br>6. Case-based learning	The basics of culture; Disparities and inequities in health; Communication in healthcare; Addressing language barriers; Role play with hospital interpreters; Healthy literacy and written language: key components to effective communication; Organizational challenges and opportunities; Immigrant health needs in pediatrics; Practical tools for pediatricians caring for immigrants and refugees; Tools for culturally and linguistically appropriate care; Working with interpreters: Providers Perspective; Interpreter roundtable Bias and racism; Racism (enhanced focus); Integration of equity in QI	2014-2020 (annually)

							7. Simulation (role play)		
Hovlid, 2022 [96]	Norway	Higher Education Institutions (HE1, HE2)	Health and social workers enrolled in postgraduate studies	ns**	QI	6 months (week-long sessions, n=4 in HE1 and 2-day sessions, n=5 in HE2)	1. Web-based module (via Zoom, *n*=1)<br>2. Didactic lectures<br>3. Group-based learning and feedback<br>4. Simulation	QI theories; Application of QI skills; Process maps; Process and outcome Indicators; Performance data analysis	ns**
Keng, 2020 [100]	USA, Canada, Spain, Greece, Romania, Brazil, Mexico	American Society of Clinical Oncology (ASCO)	Physicians-specialists (oncology)	ASCO Quality Training Program (QTP)	QI	6 months- (full course, Spain)<br><br>1-day (Canada, Greece, Mexico, Romania, Brazil and Spain)	1. Seminars (in person, *n*=3)<br>2. Case-based learning<br>3. Group-based learning<br>4. Hands-on learning at the participants' practices<br>5. Project-based learning (group project)<br>6. Project presentation<br>7. Coaching and mentorship	Introduction to QI; Problem and Aim statements; Project charter; Process analysis; Collection and Analysis of baseline/diagnostic data; Team formation and engagement; Action plan; PDSA; Evaluation and Understanding of change data; Sustainability of QI projects; Spread of QI in practice; Evaluation of outcomes; Teamwork; Facilitative leadership skills; Selection of team members and roles; Stages of group formation; Relationship-centered communication; Conflict resolution	2013-ns Updated in 2019
Kennedy, 2020 [46]	USA	University of Vermont Larner College of Medicine	Physicians-trainees (IM PGY2)	ns**	QI	10 months	1. Group-based learning (45-min sessions, *n*=10) and reflection<br>2. Project-based learning<br>3. Project presentation at local	Introduction to quality in healthcare and the IHI Model for Improvement; Affinity diagram; Process map; SMART; Baseline data collection plan; Data reflection; "5 Whys" Analysis; PDSA	2017-2018

							Quality Forum (poster)<br>4. Mentorship		
Latif, 2021 [91]	UK	Derbyshire area community pharmacy/ Leicester School of Pharmacy	Community pharmacists (working at least 15 hours per week)	ns**	QI	6 months	1. Web-based learning<br>2. Tutoring<br>3. Interactive workshops (face-to-face, *n*=3)<br>4. Group-based learning (focus groups, n=2)	Clinical pharmacy management: health policy, healthcare organisation and management; Audit techniques; Clinical governance and pharmaceutical services (training and auditing to drive service enhancement); Risk management; Dispensing and medication errors (causes, theory, investigation); Service improvement; Models of pharmacy practice; Quality management theory and performance	ns**
March, 2024 [90]	Australia	Australian Institute of Medical Simulation and Innovation (AIMSI)	Physio-therapists	MuPACT	QI, Person-centered ness	3 hours	Workshop / Interactive seminar [Simulation (Fishbowl, Role play) with professional medical actors; Debriefing; Supplementary material (PowerPoint presentation, Infographic summary, two 30-sec videos, Reflection sheet)]	Person-centered care; Acceptance and Commitment Therapy; Therapeutic alliance; Values-based goal setting; Use of metaphor	2022
Miller-Kuhlmann, 2020 [54]	USA	Stanford University	Physicians-trainees (Neurology PGY2-5)	ns**	QI	18 months	1. Didactic lectures<br>2. Group-based learning<br>3. Workshops (2 hours, *n*=3, established modules)<br>4. Case-based learning	Error analysis; Cost of care; QI project design; case presentation; Fishbone diagram; Key drivers; Possible interventions; SMART; IHI Open School; Stanford Realizing Improvement through Team Empowerment program modules	2015-2017

							5. Morbidity, Mortality, & Improvement (MM&I) conference (monthly, multidisciplinary)<br>6. Project presentation (conference, peer-reviewed journal)		
Payne, 2021 [61]	USA	Children's National Hospital, Washington, DC	Pediatric GME program directors and assistant directors	ns**	QI	18 months	1. Educational material (prework, resource manual)<br>2. Web-based modules (IHI Open School)<br>3. Group-based learning (half day workshop)<br>4. Individual coaching<br>5. Project-based learning (individual project)<br>6. Project presentation during GME committee meetings	Selected IHI Open School online modules (QI 101- 105); Systems thinking; Aim statements; SMART; QI measures; Process maps; Fishbone diagram; Failure mode and effects analysis (FMEA); PDSA; Run charts; Graphs; Understanding the system; Understanding variation; Understanding data; IRB submission; Key driver diagram; Resources for publishing QI studies	2017-2018
Pender, 2021 [62]	USA	University of Utah Health	Physicians-trainees (GS, Plastic Surgery, Otolaryngology, Urology – all levels)	High Value Care curriculum	QI, Efficacy, Person-centeredness (High Value Care)	PGY1: 1-hour sessions (n=6, monthly), PGY2: Group project	1. Didactic lectures<br>2. Group discussions<br>3. Project-based learning (group project)<br>4. Coaching<br>5. Project presentation in annual Value Symposium	Introduction to high value care and cost-consciousness; Quality in healthcare; QI principles and methods; Lean; Six sigma; DMAIC (Define, Measure, Analyze, Improve, Control); PDSA; PS; Patient experience	2016-2019

Pflipsen, 2022 [64]	USA	ns**	Faculty	Quality Improvement Faculty Development Workshop (QIFDW)	QI	4 hours	Workshop (Short lectures; Group reflection and feedback)	IHI Model for Improvement; IHI Open School modules; QI; QI measures; Aim statements; SMART; Flow chart; Cause and effect diagram; Outcome/process/balancing measures; Data collection planning; Run chart; PDSA cycle; Leading change; Spreading change; Resistance to change; QI vs Research	2016 - 2017
Pohl, 2020 [65]	USA	University of Utah	Physicians-trainees (FM PGY1-3)	Skills-based Experiential Embedded Quality Improvement (SEE-QI)	QI	3 years	1. Didactic lectures (*n*=12, annually)<br>2. Experiential learning (leading multidisciplinary QI teams)<br>3. Coaching<br>4. Project-based learning (group project)<br>5. Project presentation (oral, scholarship poster)	IHI Model for Improvement; Process definition; Process variation; Ethics of research; Decision-making tools; Process diagram; Change management; Fishbone diagram; Team functionality; Compare and contrast various QI methods; Description and implementation of the PDSA cycle; Squire guidelines; Quality improvement in professional practice; National benchmarks for quality; QI team organization; Process definition	2012-ns
Reynolds, 2021 [69]	USA	Duke University School of Nursing	Nurses (Doctor of Nursing Practice)	DNP post-doctoral Quality Implementation Scholars Program	QI	1 year (3 semesters)	1. Didactic lectures<br>2. Project-based learning (group project)<br>3. Mentorship (bi-weekly seminar, every other week with faculty members)<br>4. QI practicum (focused on implementing the QI project)<br>5. Web-based learning [online modules, Team	Data science and informatics for QI (Data and quality metrics to evaluate interventions, Data analytics); Healthcare QI methods (Run chart, Control chart, Benchmarking, Scorecards, Disseminating results, Continuous improvement); Advanced topics in health services leadership (strategic communication,<br>executive presence, interdisciplinary collaborations, principles of executive leadership and problem solving); Team based QI science initiative practicum (capacity, cost analysis, collaborative partnerships, sustainability); Completion	2019-2020

							Strategies to Enhance Performance and Patient Safety (TeamSTEPPS)]	of established TeamSTEPPS Master trainer course	
Rolita, 2022 [70]	USA	University of Hawaii	Physicians-trainees (FM), Faculty	ns**	QI	5 didactic half-days, (30min-3 hours)	1. Didactic sessions [IHI modules & videos; Group discussions and reflection]<br>2. Project-based learning (group project)	Introduction to QI; PDSA; Swiss Cheese model; Quality in healthcare; QI processes/steps; Essential elements of a QI team; RCA; PDSA cycle; Payment transformation and performance measurements; Quality of care from different actor's perspectives; Forming QI teams; QI concepts; QI goals; IHI triple aim of Population Health; Experience of Care and Per Capita cost; Clinical Data Analysis; Basic Biostatistics; Data presentation; Short QI patient surveys; Data-informed QI interventions	2018 - 2019
Schroll, 2020 [73]	USA	Tulane University School of Medicine	Physicians-trainees (GS PGY1-5) and faculty	ns**	QI	1 academic year	1. Didactic lectures<br>2. Web-based learning (IHI Open School modules)<br>3. Educational material<br>4. Group-based learning<br>5. Faculty mentorship<br>6. Project-based learning (group project)<br>7. Project presentation at a QI conference<br>8. Mentorship	QI processes; Importance of incorporating QI into healthcare systems and residency educational curricula; Types of QI projects; Adaptation from IHI Open School material (PDSA cycle, Cause & effect diagram, Fishbone diagram, Key driver diagram, Flow charts); QI toolkits and templates for developing QI project	2018-2019
Smith, 2023 [92]	UK	NHS England's (NHSE) Improvement	Health workers	Lean Fundamentals MOOC	QI	8 weeks	1. Massive open online courses (MOOC)<br>(7 modules)	Lean QI methods: Improvement kata; introduction to Lean (value, waste and PDSA); Creating standard operations for processes; The seven flows of healthcare	2021 (launched)

		Capability Building and Delivery (ICBD) team					2. Project-based learning (individual project)	(observation, documentation and process mapping); A five-step process to workplace organisation; Visual methods to design effective/efficient/reliable processes	
Sosa, 2024 [78]	USA	University of Miami	Physicians-trainees (all levels and specialties)	GME Quality Improvemen t Project Support (QuIPS) program/ curriculum	QI	4 months	1. Web-based, self-directed learning videos (*n*=4, 8-10 mins, one per month)<br>2. Virtual zoom-based interactive sessions or Hands-on workshops (n=4, one per month)<br>3. Project-based learning (group project)<br>4. Project's oral presentation (10mins)	Introduction to QI; Components of A3; Problem Statement; Background; Current Condition; Process mapping; Root cause analysis (RCA); Fishbone diagram; 5 Why's; Obtaining the voice of the customer; the "Right Side" of the A3; Key driver diagram; Plan-Do-Study-Act (PDSA) cycle; Countermeasures; Development of a data collection form; Data Visualization (Bar Charts, Histograms, Run Charts); Pareto chart; Control chart	2017-2020 (in-person), 2020- to date (virtual), biannually
Xiang, 2023 [83]	USA	ns**	Physicians-residents (IM PGY 2)	ns**	QI	5 sessions [half-day (3hr) sessions, one in every ambulat ory clinic block, every 8 weeks]	1. Didactic sessions [Case-based learning (patient vignette); Group discussion and feedback]<br>2. Project-based learning (group project)<br>3. Project Presentation to a QI expert panel and clinical leader<br>4. Mentorship and coaching<br>5. Web-based platform (meetings, QI project review)	Introduction to basic QI concepts; Institute for Healthcare Improvement (IHI) Model for Improvement; Lean Six Sigma; Gemba/Process Walk; Process map; Identification of key stakeholders; Swim Lane Diagram; RCA; Fishbone diagram; 5 Why's; Problem statements; SMART aims; IHI concept of change; Intervention action hierarchy; Effort vs Impact matrix; Data collection; Data analysis; Data visualization; Data interpretation; Run chart; Identification of structure/outcome/process/balancing measures; PDSA cycle; Sustainability; Common QI project pitfalls; Long-term QI project success	2016-2020 (in-person) 2020 –ns (virtual)

*As described by respective authors.

**Not specified.
