# Supplementary material for: What Do We Know About Contemporary Quality Improvement and Patient Safety Training Curricula in Health Workers? A Rapid Scoping Review
Source: Healthcare (Basel). 2025 Jun 16;13(12):1445. doi: 10.3390/healthcare13121445 (PMC12193159; doi:10.3390/healthcare13121445)
Supplement: Supplementary file 1 [file healthcare-13-01445-s001.zip › File S3_Narrative presentation of the 27 PS curricula.pdf]

Narrative presentation of the 27 curricula described as addressing Patient Safety (PS)-either with a conceptual approach or focusing on specific topics of PS-for graduate, postgraduate or continuous education of health workers.

**Source (First author, Year)**	**Country**	**Setting and/or developed by**	**Learners**	**Intervention's title**	**Content focus area**	**Duration**	**Teaching methods/ Structure**	**Educational content***	**Period of implementation**
Arnell, 2021 [28]	USA	Baylor College of Medicine	Physicians-Residents (IM and IM-Pediatrics PGY1-4)	ns**	PS, Event Reporting	1 hour	Workshop [Didactic lecture; Case-based learning; Simulation (mock PS event report)]	Experiences regarding PS and event reporting; Key PS terms and concepts; Defining medical error/near misses/preventable events; PS event rates literature review; Life cycle of a PS event report; RCA and peer review; The value of reporting by frontline providers and legal protection for reporters; I-SAFEST framework; Fictional case involving a delay of antibiotic administration	2019
Bagian, 2023 [29]	USA	University of Michigan Center for Healthcare Engineering and Patient Safety	Physicians-residents (Anesthesiology, Orthopedic Surgery, Ophthalmology)	Root Cause Analysis and Action (RCA^2) training program	PS	ns**	1.Web-based learning (interactive training modules, *n*=6)<br>2. Workshops (Face-to-face, *n*=6)<br>3. Group-based learning<br>4. Case-based learning<br>5. Project-based learning (group projects)<br>6. Project presentation (30 mins, during departmental grand rounds)<br>7. Intersession work (record/policy/procedure/publication	Human Factors Engineering: The basic science of PS; RCA^2: The process; RCA^2: Event Story Map; RCA^2: Cause and Effect Diagramming; RCA^2: Five Rules of Causation; RCA^2: Actions and Outcome measures;<br>Action analysis; Flow diagram of initial understanding; Identifying information needed to understand what/why it happened; Conducting interviews; Accomplishi=g document; record; and literature reviews; Identifying root cause and contributing factors (RCCFs)	2013-2019

							review, stakeholder interviews)		
Beaumont , 2020 [85]	Canada	Canadian Society of Otolaryngology Head and Neck Surgery annual meeting	Physicians-residents (Otolaryngology, all), staff, fellows	Peri-Operative Patient Safety	PS, Peri-Operative Patient Safety	2 hours	Workshop [Didactic lecture Case-based learning Group-based learning and discussion]	Duty of care; Liability; Regulatory authorities; Procedural error with regards to NOTSS (situational awareness, decision-making, communication/teamwork, and leadership); Follow-up management; Barriers to follow-up and resolutions	2017
Brown, 2020 [88]	Australia	Large metropolitan Australian university	Nurses (graduate entry master's students)	Master of Nursing Practice GEM program	PS	3 semesters	1. Didactic lectures 2. Simulations 3. Group-based learning, discussion and debriefing	Fundamentals of nursing; Orientation; Communication; Therapeutic relationship building; Admission to discharge nursing care of an uncomplicated surgical patient: Admission; Preoperative care; Postoperative care: Removal of intravenous catheter, Indwelling Urinary Catheter, and wound drains; Discharge; Behavioural and mental health care: Auditory hallucinations and hearing voices; Communication with emotionally disturbed persons in the emergency department; Communication with persons experiencing thought disorder (delusional ideation and form of thought disorder); Communication with persons experiencing suicidal and self-harm ideation; Communication with highly anxious persons; Complex nursing care: Cardiac (Nursing care of a patient after angiogram, nursing care of a patient pre-,	2014- ns**

								intra-, and post-cardioversion); Respiratory (Nursing care of a patient after thyroidectomy, nursing care of a patient with acute asthma); Neuro and trauma (Nursing care of a patient with an acute head injury after fall from height, nursing care of a trauma patient cyclist vs motor vehicle); Blood and burns (Nursing care of a patient presenting with acute face and hand burns, nursing care of a patient experiencing postpartum haemorrhage); Advanced life support; The roles of nurses in Advances life Support; Identify–situation–observations–background–agreed plan–read back (ISOBAR)	
Fox, 2021 [38]	USA	Children's Hospital of Pittsburgh	Physicians-residents (Pediatrics, IM-Pediatrics, and triple board: pediatrics, adult psychiatry, and child and adolescent psychiatry)	ns**	PS	ns**	1. Didactic lectures<br>2. Workshops and forums (annual for senior residents)<br>3. Error-reporting system<br>4. Experiential learning (PS behaviors integrated in daily activities)<br>5. Case-based learning (monthly sessions for interns and senior residents)	PS concepts; Identification of factors leading to errors; Culture of safety; Just culture; Importance of event reporting; Easy-to-use event-reporting system (RISKMASTER Accelerator); Error disclosure and accountability; RCA; Interdisciplinary case presentation; Identification of interventions for preventions; Emphasis on PS culture on daily rounds and in morning sign-out from the night team; Participation of Chief residents in daily hospital Safety call	2010-2015

Frenzel, 2020 [39]	USA	North Dakota State University School of Pharmacy	Pharmacists, Pharmacy technicians	ns**	PS	105 min	Workshop [Didactic lectures (45min); Simulations (20min, *n*=3); Multimedia (audio recordings); Group-based learning, discussion and debriefing]	Overview of medication errors; PS; Continuous QI; Identification of types of medication errors in institutional and community pharmacy; RCA to identify contributing factors; Fishbone analysis; Strategies to reduce medication errors rate; Resources available to pharmacists and patients regarding the identification and prevention of medication errors; North Dakota pharmacy statute on continuous quality improvement	ns**
Gross, 2022 [41]	USA	Mott Children's Hospital	Physicians-residents (Pediatrics, Emergency medicine, Family Medicine), Physician Assistants	ns**	PS, Handoff	ns	1. Didactic lectures (in-person, during conferences, resident-led)<br>2. Web-based learning (virtual lecture)<br>3. Multimedia [video, I-PASS (Illness Severity, Patient Summary, Action List, Situation Awareness & Contingency Planning, Synthesis by Receiver) poster in clinician work areas)<br>4. Simulation	Survey data on current handoff state; I-PASS tool; Video simulations of handoffs utilizing I-PASS	2018
Harris, 2020 [43]	USA	University of Kentucky College of Medicine, Hospital of the	Physicians-residents (urology), faculty	ns**	PS	6 months	1. Didactic lectures (*n*=2, Adapted modules from Jeffrey Oakley's "Accident Investigation	Accident theory; Tools to assess adverse events; Events and causal factors analysis; Change analysis; Barrier analysis; Tree analysis; Fishbone analysis; 5-why's; RCA	ns**

		University of Pennsylvania					Techniques" and the American College of Surgeons quality in-training initiative)<br>2. Case-based learning<br>3. Group presentation and discussion<br>4. Morbidity and Mortality (M&M) conference<br>5. Project-based learning<br>6. Project presentation at M&M conference	techniques; Case analysis from M&M conference	
Kim, 2022 [48]	USA	University of California, Los Angeles David Geffen School of Medicine	Physicians-surgery cardiothoracic surgery residents (PGY6-7)	Non-Technical Skills for Surgeons (NOTSS) curriculum	PS, Non-technical Skills	1 month	1. Didactic lectures<br>2. Group-based learning, discussion and debrief<br>3. Multimedia (Brigham and Woman's Hospital videos)<br>4. Self-learning (book)<br>5. Simulation (4 15min case vignettes in a simulated operating room)	NOTSS concepts (Brigham and Woman's Hospital video highlights): Situational awareness (gathering information, understanding information, projecting and anticipating future state); Decision-making (considering options, selecting and communicating option, implementing and reviewing decisions); Communication and teamwork (exchanging information, establishing a shared understanding, coordinating team activities); Leadership (setting and maintaining standards, supporting others, coping with pressure); Group debrief; Videos on good and poor nontechnical skills in the operating room; Copy	2018 (first 4 residents), 2020 (last 4 residents)

								of the book "Enhancing Surgical Performance: A Primer in Non-Technical Skills"	
Kim, 2023 [47]	USA	US Department of Veterans Affairs Boston Harvard South Shore Psychiatry Residency Training Program	Physicians-residents (Psychiatry, all)	Patient safety presentati on (PSP)	PS	1 hour	1. Didactic lecture (in-person, resident-led)<br>2. Case-based learning	PS; Event reporting; Summary on healthcare system's recent PS reports and investigations; RCA; Introduction to relevant literature	2021
Lim, 2021 [98]	S. Korea	Hanyang University	Nurses	Cases for Accounta bility and Safety Enhance ment (CASE)	PS	5 weeks	1. Web-based learning (15 min modules, *n*=9)<br>2. Multimedia (Cartoons)<br>3. Case-based learning	Nurses' Legal obligation and responsibilities (civil liability, criminal liability, administrative punishment); Medical malpractice; PS incidents; PS competency enhancement activities; RCA; Analysis of malpractice cases from the Korean Supreme Court: Main issue; Overview; Violation of legal obligations; Nurses' legal responsibility; RCA; Enhancement activities for PS competency	2019
Maeda, 2022 [97]	Japan	Jichi Medical University	Physicians-residents	ns**	PS, incident report writing	1 hour	1. Didactic lectures<br>2. Multimedia (already established video)<br>3. Simulation (incident reporting - IR)<br>4. Group-based learning and feedback	Introduction to incident reports (IR) (purpose, definition, and application); Fictional incident video; Creation of a freestyle simulated IR; Peer evaluation among participants; Fact description method (When/Where/How did the event occurs, Who, What did you see/hear/say); Fictional incident video (same); Creation of a simulated IR based on the description method; Examples of fact descriptions	2021

Mu, 2021 [55]	USA	Midwestern Veterans Affairs (medical center	Physicians, Graduate-degree Nurses, Pharmacists	Interprofessional patient safety fellowship program (former expanded Chief Resident in Quality and Safety training)	PS	1 year	1. Didactic lectures (in person/virtual)<br>2. Web-based learning (Institute for Healthcare Improvement (IHI) Open School modules, biweekly)<br>3. Project-based learning (group project)<br>4. Mentorship<br>5. Journal club (biweekly)<br>6. Project presentation (professional conferences, manuscript draft and submission, grand rounds)<br>7. Group-based learning and decision-making<br>8. PS elective	Attendance of Chief Resident in Quality and Safety (CRQS) or National Center for Patient Safety (NCPS) orientation/boot camp; Attendance of a month-long patient safety elective through the affiliated Medical College; Team building; CRQS or NCPS virtual training on PS/journal club; IHI Open School modules (QI 101–Q105, PS 101–105, TA 101, PFC 101, and L 101); Lean training	2011-ns
O'Toole, 2020 [58]	USA	I-PASS Study Education Executive Committee, Society of Hospital Medicine (SHM)	Physicians-frontline providers (resident, hospitalists, advanced practice providers, and fellows)	SHM I-PASS Mentored Implementation Handoff Curriculum	PS, Handoff	90 minutes	1. Independent pre-session work (Patient summary and written handoff exercises)<br>2. Workshop (in-person, 90min) [Simulation; Case-based learning; Multimedia (Video modules); Educational material (printed documents,	I-PASS introductory video: main components background; mnemonic; TeamSTEPPS techniques; Handoffs vignettes (poor communication vs team-based); Development of effective patient summary in the I-PASS structure; Handoff simulation exercises	2015-2016

							workshop slides); Group-based learning and discussion]		
Paull, 2023 [60]	USA	Accreditation Council for Graduate Medical Education (GME)	GME institutional officials, GME program directors, PS/Quality Improvement (QI)/risk management staff, executive leaders (Chief medical officer, Chief nursing officer), residents (PGY1), fellows	Pursuing Excellence Initiative (PExI) Pathway Leaders Patient Safety Collaborative	PS	18 months	1. Didactic lectures (in-person, *n*=2)<br>2. Web-based learning (virtual sessions, monthly, *n*=9)<br>3. Group-based learning, discussion and debriefing<br>4. Coaching and Check-in Calls<br>5. Intersession work	Rules of causation; Writing causal statements; Understanding strengths of various actions in preventing event recurrence; RCA and RCA^2 frameworks; PS champions; Stakeholder analysis; Human Factors Engineering principles; Quantifiable outcome measures; Leadership concurrence; Flow diagrams; Event story maps; Cause-and-effect diagrams; Strong causal statements; Elements of effective PS event analysis; Close calls for PS event investigation; Strong String Assessment Tool to evaluate the quality of a PS event investigation; COVID-19 PS events; Principles of proactive analysis of organizational threats; Sustainability for QI projects; Visual management boards	2017-2021
Perkons, 2024 [63]	USA	Thomas Jefferson University Hospital	Physicians-residents [Internal Medicine (IM) PGY1)	ns**	PS	2 weeks	1. Web-based learning (30 min module, independent prework)<br>2. Group-based learning (3 hours)<br>3. Case-based learning<br>4. Independent intersession work (1 hour, record/chart/policy/g	PS Definitions (medical error, adverse event, near miss, active failure, latent condition, root cause); Responding to medical errors; Swiss Cheese model; Safety Event Review; Root cause analysis (RCA); Apparent cause analysis; Systems issues contributing to the error; Cause and Effect diagram; Flow chart/event timeline/story map; Causal thread; 5 Rules of	2020-2021

							uidelines/publication review, stakeholder interviews)<br>5. Presentation of investigation and proposed action plans to the Hospital Medicine PS Committee (30 mins)	Causation; Identification of actions for improvement	
Quinones Cardona, 2021 [67]	USA	Academic urban children's hospital in Philadelphia, Division of Neonatology, Department of Pediatrics, St Christopher's Hospital for Children, Drexel University College of Medicine	Physicians-residents (neonatal, pediatrics PGY2 & attendings, Nurses, Physician assistants	NICU I-PASS (Illness Severity, Patient Summary, Action List, Situation Awareness & Contingency Planning, Synthesis by Receiver)	PS, Handoff	ns**	1. Training session (Simulations; Group-based learning)<br>2. Presentation to staff by nursing champions (weekly)<br>3. Newly designed electronic handoff tool<br>4. Data display in work and conference rooms	I-PASS training session; Plan-Do-Study-Act (PDSA) cycles; Literature review; Presentation of interruptions to nursing and hospital administrative leadership biweekly; Avoidable versus unavoidable interruptions	2015-2018
Reilly, 2024 [68]	USA	Penn State Hershey Pediatric Residency	Physicians-residents (Pediatrics)	Stats, Assessment, Focused plan, Exam, To do, If/then, Pointers/pitfalls, Sick-o-meter	PS, Handover	ns**	1. Didactic lectures<br>2. Simulated practice sessions	SAFETIPS ("S"tats, "A"ssessment, "F"ocused plan, "E"xam, "T"o do, "I"f/then, "P"ointers/pitfalls, "S"ick-o-meter)	2016-2018

				(SAFETIPS)					
Schall, 2022 [72]	USA	Brooke Army Medical Center	Physicians-residents/fellows, GME faculty, non-physicians	RCA W3 (What happened? What should have happened? What are you going to do to fix it?)	PS	8.5 hours	1. Didactic lectures<br>2. Group-based learning and discussion<br>3. Simulation (actor interviewing)<br>4. Case-based learning<br>5. Web-based learning (online modules, National Patient Safety Foundation modules, Veteran Affairs National Center for Patient Safety resource guide)<br>6. Multimedia (Air Force Medical Operations Agency & Brooke Army Medical Center Healthcare Resolution Specialist videos)	RCA²: Improving Root Cause Analyses and Actions to Prevent Harm; Poor versus good RCA interviewing technique; DHA Root Cause Analysis: Partnering for a New Level of Care Course; RCA resource guide; Disclosing medical errors to patients and their families; Fundamentals of PS science; RCA principles; Preliminary data gathering and organization; Identifying causal factors; Drafting causal factor statements; Developing corrective action plans	2018-2020
Seitz, 2023 [74]	USA	Internal Medicine Residency Program, University of Washington School of Medicine	Physicians-residents (IM Chief residents)	Near-Peer Support Framework	PS, Trainee Well-Being after Patient Safety Events	2 hours	Workshop [Case-based learning; Simulation (vignettes); Group-based learning]	Outreach; Confidentiality; Opening; Listening; Reflection; Reframing; Normalization; Sense-making; Acknowledgement and thank; Pause and coping; Resources and referrals; Follow up; Resident error and patient harm; Unexpected event (process overlapping emotions); Grieving Loss	

Shrivastava, 2022 [75]	USA	Donald and Barbara Zucker School of Medicine at Hofstra/Northwell	Physicians-residents (IM), medical students, clinical pharmacy/physician assistant graduate students, health psychology trainees	ns**	PS, Emotional intelligence (EI) and communication skills in Interprofessional Leadership Development	2.5 hours	1. Workshop<br>2. Didactic lectures<br>3. Group-based learning, activity and reflection<br>4. Multimedia (video)<br>5. Self-assessment (emotional intelligence and communication styles)	Interprofessional Education Collaborative competences; EI self-assessment tools; Communication style inventory; Skills required for great leadership; Leadership skills; Importance of EI and communication in leadership; Key components of EI; Review and discussion on own EI scores; Identification of EI strengths; weaknesses and components to be improved; Communication styles; Communication challenges with difficult team members; Understanding team dynamics (fishbowl activity); Importance of communication and EI skills for teamwork and leadership	
Sloane, 2020 [76]	USA	Nursing Homes (NH)	Physicians, Nurse Practitioners, Physician Assistants	ns**	PS, Antibiotics Stewardship	18 months	1. Web-based learning (2-hour video training for nursing staff, *n*=10)<br>2. Multimedia (1-hour training CDs *n*=2; Informative posters changed on a quarterly basis; Pocket information and reminder cards; Information brochure for NH residents and families)<br>3. QI newsletters (quarterly)	Case examples of common issues in antibiotic stewardship; Antibiotic stewardship; Urinary tract infections; Respiratory tract infections; Skin and soft tissue infections; SBAR; Common reasons for antibiotic overprescribing; Algorithm regarding recommendations for when to obtain urine cultures; Guidelines for gathering clinical information before contacting on-call medical providers; NH infection control guidelines; Antibiotic stewardship for residents and families	2015-2017

							4. 1-year QI poster for nursing staff<br>5. Case-based learning		
Swanson, 2021 [79]	USA	Community-based Radiation Oncology facility	Physicians-radiation oncology, Medical physicists, Radiation therapists, Nurses, Support personnel	Customized Crew Resource Training	PS, Error reporting	6 months	1. Didactic lectures (45min, *n*=2, weekly)<br>2. Case-based learning<br>3. Multimedia (video)	Radiation oncology industry best practices; Adverse events; High-reliability organization principles; Informally reported "close calls" within the department; Case studies; Methods and tools for incident recognition; Characterization; Reporting criteria; Team-based safety and risk mitigation strategies (leadership, situational awareness and assertiveness); Structured communication techniques; Team-based safety protocols; Team Strategies to Enhance Performance and Patient Safety (TeamSTEPPS); Situation, Background, Assessment, and Recommendation (SBAR); Joint Commission RPI Model; Web-based customized RO incident learning system	2016
Vijayan, 2022 [80]	USA	Valley Children's Hospital	Physicians-residents (Pediatrics)	ns**	PS	30 months	1. Didactic lectures<br>2. Experiential learning (resident-led PS event reporting)<br>3. Group-based learning and feedback	PS; PS event (PSE) reporting; Well-written PSE reports; Clinical learning Environment Review pathways; Electronic reporting system demonstration; Specific, Measurable, Achievable, Relevant, and Time-Bound (SMART); Role of residents in safeguarding PS;	2018-2021

								Consensus building among stakeholders to include residents in PS; Creation of resident generated PSEs; Discussion of resident generated PSE reports; Actions taken by PSEs and dissemination thereof; Development of interventions to address PSE; Inclusion of risk reduction strategies in PSE reporting	
Wahlberg, 2022 [81]	USA	University of Vermont Medical Center	Physicians-residents (IM PGY3)	ns**	PS	5 hours (1-hour sessions, *n*=5, during a 4-month period)	1. Didactic lectures<br>2. Group-based learning<br>3. Case-based learning<br>4. Simulation (mock RCA)	Introduction to PS principles; Just culture; Institutional PS reporting system review; Common causes of error; System error; Performance error; Cognitive bias; Review of near miss resident-reported events; PS reporting; Event identification and investigation; Role and responsibility reviews for mock RCA; mock RCA analysis; Recommendations for systems change	2017-2020
Wallace, 2022 [82]	USA	University of Florida, University of Rochester	Physicians-fellows (Hematology/Oncology)	ns**	PS	2 hours	1. Didactic lectures (1 hour, *n*=2)<br>2. Case-based learning<br>3. Group-based learning<br>4. Simulation (vignette, actor interviews, mock RCA)	RCA structure and process; QI tools; Flow diagram; Fishbone diagram; Swiss cheese model; Root cause/contributing factor statements	2017, 2020
Ziemba, 2021 [84]	USA	Hospital of the University of Pennsylvania	Physicians-residents, Nurses,	Root Cause Analysis (ReCAst)	PS, Root Cause Analysis Simulation	1 academic year	1. Simulation (90min, *n*=11)<br>2. Case-based learning	Individual knowledge/behavior assessment; How to submit an adverse event; RCA; Adverse event report review;	2019-2020

			Pharmacists, Therapists	Simulation Program			3. Group-based learning and discussion<br>4. Individual reflection	Interviews/Timelines/Equipment; Fishbone diagram; Hierarchy of Systems Design; Improvement solutions; Systems redesign; Report out	

*As described by respective authors.

**Not Specified.
