# Supplementary material for: What Do We Know About Contemporary Quality Improvement and Patient Safety Training Curricula in Health Workers? A Rapid Scoping Review
Source: Healthcare (Basel). 2025 Jun 16;13(12):1445. doi: 10.3390/healthcare13121445 (PMC12193159; doi:10.3390/healthcare13121445)
Supplement: Supplementary file 1 [file healthcare-13-01445-s001.zip › File S4_Narrative presentation of the 20 QIPS curricula.pdf]

Narrative presentation of the 20 curricula described as addressing Quality Improvement and Patient Safety (QI/PS) for graduate, postgraduate or continuous education of health workers.

Source (First author, Year)	Country	Setting and/or developed by	Learners	Curriculum's/ program's title	Content focus area	Duration	Teaching methods/ Structure	Educational content*	Period of implementation
Chen, 2021 [32]	USA	University of Washington Internal Medicine (IM) Residency Program, Harborview Medical Center	Physicians-residents (IM PGY1-3)	ns**	QI/PS	3 years	1. Seminar (PGY1-half day, *n*=1/ PGY2, half day per week, *n*=4)<br>2. M&M conference (PGY2-3, biweekly)<br>3. Project-based learning (PGY2-3)<br>4. Mentorship/Coaching<br>5. Reporting PS events (experiential learning)<br>6. Case-based learning<br>7. Group-based learning<br>8. Presentation of findings to medical center QIPS leadership<br>9. Active reflection	PS culture; QIPS Methods; Investigating PSE cases; Reinforcing PS culture and just culture; FMEA; Process map; Hazard analysis; RCA methodology (chain of events, fishbone diagram, stakeholder interviews, Gemba walks, cause map analysis, causal statements); Lean; Six Sigma; IHI Model for Improvement; Data analysis (run charts, statistical process control); Aim statements; Measures; Designing interventions (strength, impact–effort analysis); Teams; Charters; PDSA	2015-2018
Chen, 2023 [33]	USA	University of Washington, Veterans Affairs	Physicians-residents [Internal medicine (IM)	ns**	QI/PS	Half-day (PGY1)<br><br>4 weeks (1 half-day	1. Web-based learning (synchronous)<br>2. Workshop	Culture of PS; High-reliability organisation principles; Proactive identification of possible future harms; PS event (PSE) reporting; PSE case investigation; QI methodology; Process/Cause maps; Impact/effort matrixes; Design	2020-2021

		Puget Sound Healthcare System	neurology, ophthalmology PGΥ1, IM, FM PGY2]			weekly, PGY2)	3. Group-based learning (small groups)<br>4. Didactic lectures (large groups)<br>5. Case-based learning<br>6. Mentorship<br>7. Project-based learning (group project)<br>8. Presentations to medical center leadership	measures/project charters; Stakeholder interviews; PDSA	
Durstenfeld, 2020 [37]	USA	New York University School of Medicine	Physicians-residents (IM PGY2)	ns**	QI/PS	2 weeks	1. Workshops (up to 3 hours, *n*=8)<br>2. Project-based learning (group project)<br>3. Case-based learning<br>4. Group-based learning, discussion and feedback	Lean Training; Charter construction; Process mapping; Problem identification; Solution design; Introduction to the Rotation/Choosing a Problem; Value-Based Medicine; Metrics; Dashboards; Health Informatics; Systems thinking case study; Antibiotic Stewardship; History of PS; High Reliability Organization Principles; Transitions of Care; Shark Tank Pitches; Stakeholder interviews; Ishikawa diagram	2017-2018
Goldstein, 2024 [40]	USA	The University of Texas McGovern Medical School	Physicians-residents [Family medicine (FM)]	ns**	QI/PS	1 academic year	1. Didactic lectures (*n*=3)<br>2. Web-based learning [self-paced, American Academy of Family Physicians (AAFP-TIPS QI) modules]<br>3. Morbidity & Mortality (M&M) conferences	Principles of QI; Distinguishing QI from human subjects research; Institute of Medicine (IOM) six aims for healthcare improvement; AAFP-TIPS QI; System thinking; Root cause analysis (RCA)	2020-2021

							(quarterly, cases discussion followed by a 20-minute teaching session)<br>4. Case-based learning<br>5. Project-based learning (individual project, PGY 2)<br>6. Project presentation		
Hulett, 2020 [45]	USA	Elmhurst College	Nurses (postgraduate students)	Master of Science in Nursing (MSN) program	QI/PS	ns**	1. Web-based learning (IHI Open School modules)<br>2. Didactic lectures	IHI Open School modules (QI 101–Q105, PS 101–105, TA 101, PFC 101, and L 101)	2017-2019
Kurup, 2020 [49]	USA	Mayo Clinic	Physicians-residents (radiology PGY3)	ns**	QI/PS	1 academic year	1. Didactic lectures (hour - long monthly)<br>2. Peer-to-Peer Teaching and feedback<br>3. Web-based learning (IHI Open School modules)<br>4. Project-based learning (group project)<br>5. Project publication in peer-reviewed journals	Application of QI methodology through tangible problem solving; Define the problem; Measure, Analyze, Improve the root causes; Lean Six Sigma;<br>DMAIC (define, measure, analyze, improve, and control); Control and sustain the gains; Final report outs; Noninterpretive skills board review; Hand off to new residents; IOM reports; Human factors; High-reliability organizations; Human-factors engineering; Standardization; Communication; Routine reporting; Nonstandard communication expectations; Culture of safety; Authority gradient; Just culture; Manageable behaviors; Human error; Diagnostic error;<br>Procedural complications and morbidity; Periprocedural care; Patient identification and assessment; Informed consent and minors' rights; Universal protocol; Sedation; Medication	2014- ns**

								reconciliation; Hand hygiene; RCA; Fishbone diagram; Pareto chart; 5 Whys; Active error vs latent condition; Daile management systems; Huddles; short video “Realizing Improvement through Team Empowerment” (RITE) tutorials; SMART goal statements; Identifying stakeholders/physician sponsor/process owner; Data location/collection plan; Sample size; Baseline data collection; PDSA; Graph and assess pre- and postintervention data; Control plan; Summarize project activities, outcomes and outstanding barriers or issues; Celebrate learnings and project outcomes;	
Lee, 2023 [50]	USA	University of Southern California	Physicians-fellows, faculty members	ns**	QI/PS	18 months	1. Didactic sessions (1hr, monthly, hybrid format)<br>2. Simulation<br>3. M&M conferences (monthly)<br>4. Project-based learning (individual project)<br>5. Project presentation (quarterly updates)	Human factors; Cognitive biases and assumptions; Variability in processes that lead to systems failure;<br>QI Tools from Agency for Healthcare Research and Quality (AHRQ); Plan-Do-Study-Act (PDSA) cycle; Team Strategies to Enhance Performance and Patient Safety (TeamSTEPPs); Communication practices; AHRQ Briefing and debriefing tools	2019-ns**
Levy, 2023 [51]	USA	University of Michigan	Physicians-residents (IM, Medicine-Pediatrics-PGY1-2)	ns**	QI/PS	2 years	1. Didactic lectures (1.5 hours, PGY1, *n*=2)<br>2. Case-based learning<br>3.Project-based learning (group project, PGY2, 4 weeks, 58 hours)	Lean methodology; PS culture; Problem Selection; Project scoping; A3 overview; Background; Current state; Problem statement; Goal development; RCA; Countermeasures; Recommendations; Interpretation; Project Stakeholders; Sustainability; PDSA; Reflections; Lessons learned	2009-2016, 2017-2020

							4. Educational modules-flipped classroom model (PGY2, weekly, *n*=4)<br>5. Continuing work on QI/PS project after the 4-week duration (optional)		
Lin, 2020 [52]	USA	Veterans Affairs hospital, Spinal cord injury center, Community living center	Physicians-residents (IM PGY≥2)	Resident Inpatient Training Experience (RITE)	QI/PS	1 month	1. Self-learning<br>2. Facilitated discussion (group-, case-based learning)<br>3. Experiential learning (ward rotation)	Basic PS Principles; Extent and cost of medical errors in the US; Swiss cheese model; QI; Hospital Metrics/Reimbursement; Cost-Conscious Care; IOM's six aims for QI; IHI Model for Improvement; PDSA; Physician Billing and Coding; Transitions of Care; Obstacles in the discharge process; Strategies to develop hospital QI initiatives and necessary criteria to qualify for different post-acute care facilities	2013-2014
Luty, 2022 [53]	USA	Oregon Health & Science University	Physicians-residents	ns**	QI/PS	8 hours	1. Web-based learning<br>2. Multimedia (eight 5-10min videos)<br>3. Self-learning (pre-session readings and videos)<br>3. Didactic lectures<br>4. Simulation sessions (*n*=4)<br>5. Group-based learning, discussion, debrief and reflection	IHI Model for improvement; American Medical Association health system science framework; Deming's System of Profound Knowledge; Growth mindset and health system improvement; Communication errors; Best practice healthcare team dynamics; Provider burnout and well-being support sources; Institute of Medicine (IOM) Crossing the Quality Chasm framework six aims; Culture of Safety; Just/Reporting/Informed/Learning Cultures; Lean methodology; Error disclosure and reporting; RCA; Human factor engineering; Iterative implementation of an action plan; Consolidation of lessons learned into routine operations with Lean huddles; Aim statements; PDSA cycles; Types of measures; Run chart; Types of waste; A3 problem solving; Informant interviews; Fishbone; RCA statements; Action	2018

							6. Individual reflective activities	plan; Iterative improvement methods (aims, measures, ideas for change, cumulative PDSA testing); Lean process and tools (A3 thinking, tiered huddles, 5S, improvement events); Team daily management & weekly improvement huddles	
Murray, 2023 [93]	Ireland	Royal College of Physicians of Ireland	Physicians-residents	QI Scholar in Residence Programme	QI/PS	1 year	1. Mentorship<br>2. Peer-to-peer learning<br>3. Graduate quarterly meetings<br>4. Group meetings with field leaders<br>5. Conference attendance and presentations<br>6. Project-based learning (individual project)<br>7. Practice-based learning	Specific, Measurable, Achievable, Relevant, and Time-Bound (SMART) aims; Incorporating interventions into routine workflow; Run charts	2016
Mustafa, 2020 [56]	USA	University of Missouri-Kansas City	Physicians-residents (IM PGY1-3)	QIPS CURE (Quality Improvement and Patient Safety Curriculum and Resident Experience)	QI/PS	1 year (2.25 hours every 5 weeks)	1. Didactic lectures<br>2. Web-based learning (IHI Open School modules and videos)<br>3. Workshops<br>4. Project-based learning (group projects, annually)<br>5. Simulation	IHI Open School modules; IHI Model for Improvement; IHI essentials toolkit; Simulated RCA; PSE reporting; Out/inpatient quality metrics; SMART aim; Process map; Flow chart; IHI game 'How to measure a banana'; PDSA; Compiling/analyzing/interpreting QI data; PSE simulation; SQUIRE guidelines	2014- ns**

							6. M&M conferences<br>7. Group-based learning and feedback<br>8. Project presentation at annual Quality and PS day<br>9. Mentorship and coaching<br>10. Games		
Neumeier, 2020 [57]	USA	University of Colorado	Physicians-fellows (adult and pediatric subspecialties)	Fellows' Quality and Safety Academy	QI/PS	3 seminars repeated biannually	1. Seminars<br>2. Didactic lectures<br>3. Group-based learning<br>4. Case-based learning<br>5. Coaching<br>6. Simulation<br>7. M&M conferences<br>8. Project-based learning	Foundations in PS; Define Adverse PSE and Classify Levels of Harm; Common themes involving error; Level of harm; RCA tools to Analyze System-Based and Cognitive Error; Fishbone diagram; Types of cognitive biases; 5 Why's; Process map; Cause and effect diagram; Involving Stakeholders; Creating Learning Opportunities; Facilitating Discussion; Stakeholder map; Learning objectives; Principles of just culture; Facilitation tips; Identify Actionable Safety Issues; Action priority matrix (effort vs impact); Pareto chart; Current QI initiatives; Mock M&M Conference; Adverse Events Into QI; Define and Understand Problem and Identify Areas of Improvement; Gemba walk; Aims Statement; SMART goal; Measuring Progress; Structure/process/outcome/balancing measure; Effective and Reliable Improvements; Hierarchy of intervention effectiveness; Education interventions; Electronic medical record (EMR) interventions; Forced functions; Data collection for Measuring Impact; Hospital/EMR-based data vs public data; Learner resources to request data; Quality in Academics: Creating a Climate for Change; Establishing urgence/motivation; Building	2017- ns**

								coalitions and guiding teams; Creating vision for change Engaging; Implementing and Sustaining Change; Empowering colleagues; Communicating vision; building credibility and culture; QI and the IRB; IRB submission guidelines; Scholarship in QI: Publication and Grants; SQUIRE guidelines; Review of example publication; Local and national grants	
Patterson, 2022 [59]	USA	University Health	Health workers, academic faculty and staff	Clinical Safety & Effectiveness (CSE) course	QI/PS	ns**	1. Didactic sessions (*n*=7, across several months)<br>2. Project-based learning (team project)<br>3. Group-based learning<br>4. Coaching<br>5. Web-based platform<br>6. Project presentation to stakeholders, sponsors and team members	IHI Model for Improvement; Healthcare Quality in the US; QI; PS; QI tools (Aim statement, Process flow, Team effectiveness); Data variation; Data collection; Quality and cost relationship; Adaptive leadership; Lean methodology; RCA; FMEA; Team STEPPS; PDSA cycle; Flow chart; Cause effect diagram; Building safety culture: lessons from aviation; Measuring error in health care; Alumni QI project presentation; Return on investment	2005
Pensieri, 2023 [95]	Italy	Fondazione Policlinico Universitario Campus Bio-Medico, Rome	Physicians, Nurses, Healthcare technicians, Biologist, Physical therapists, Social health workers, Pharmacists, Administra	Campus Game	QI/PS	9-13 weeks	Gamification	Occupational Health and Safety; Infection Control; Privacy; Public Relations; Human Resources; Hospital Social Service; Quality and Clinical Risk Management	2021

			tive support workers						
Poonia, 2021 [66]	USA	University of Pennsylvania	Physicians-residents (Otorhinolaryngology, PGY1-5)	ns**	QI/PS	ns	1. Web-based learning (IHI Open School modules)<br>2. Group-based discussion and learning (resident-driven “Resident Safety Huddle” meetings, *n*=10, monthly)<br>3. Didactic lectures	IHI Open School modules (QI 101–Q105, PS 101–105, TA 101, PFC 101, and L 101); Introduction to QI and PS; Open discussion of safety concerns; Characterization of the problem; Analysis of the current condition; Brainstorming potential solutions; Completion of a PSE report	2018-2019
Ruiz Colón, 2023 [71]	USA	Stanford University	Physicians-residents and fellows	Safety Quest	QI/PS	4 case-based, progressive levels	Gamification	Introduction to safety and QI basics: Best practices, Goals of care (GOC) communication techniques [I-PASS handoffs, Situation-Background-Assessment-Recommendation (SBAR) communication, stop the line, call for help early, debriefing, GOC documentation); Apply QI tools and concepts (PDSA, A3, high reliability, “Swiss Cheese”)<br>Moving beyond the basics: Systematic learning from error (5 Whys RCA, Pareto Curve, Fishbone/Ishikawa Diagram) Utilizing best practices as medicine reconciliation; Order sets and checklists; QI tools (process mapping and systems approach principles); Key safety and teamwork concepts to promote positive safety culture<br>Implementing QI: Advanced QI tools [Failure mode and effect analysis (FMEA), stakeholder analysis, A3 key drivers, project sustainability); Performing time-out/Universal Protocol to prevent sentinel events/never events (e.g. wrong	2015 (created)<br>2018-ns (incorporated in curricula)

								site, wrong procedure, and wrong person surgery, retained foreign objects); Event and near misses reporting and error disclosure to patients and families<br>Mastering QI: Lean principles (5 Ss and reducing/eliminating waste (3 Ms); QI data analysis using statistical process control; High value care principles in practice	
Sanford, 2021 [99]	Japan, Spain, Sweden, South Africa, UK, USA	International Network of Universities consortium	Nurses (undergraduate and graduate nursing students, completed at least 2 years of a baccalaureate equivalent nursing program), faculty	Quality and Safety Education for Nurses (QSEN)	QI/PS	10 days	1. Workshop<br>2. Didactic lectures<br>3. Experiential learning (field observations, discussion with PS experts)<br>4. Simulation<br>5. Case-based learning<br>6. Group-based learning and discussion<br>7. Reflective learning and writing<br>8. Games<br>9. Multimedia (video)<br>10. Project-based learning (team project)<br>11. Teambuilding exercises<br>12. Web-based learning	Systems thinking; Reflective practices; Person-centred care; Safety science; Interprofessional teamwork and communication; Impact of healthcare systems/innovation/education/environment/culture on PS and quality of care	ns**
Society for Maternal-	USA	Society for Maternal-Fetal	Physicians-fellows (maternal	Curriculum on	QI/PS	3 years	1. Web-based learning (online modules,	Definitions of direct/indirect supervision and oversight; Culture of safety; Care transitions; Structured Handoffs; Teamwork; I-PASS; I-	2023

Fetal Medicine, 2023 [77]		Medicine (SMFM)	fetal medicine-MFM), MFM fellowship program directors	Patient Safety and Quality for MFM fellows			established content, one topic per month)<br>2.Project-based learning (individual project)<br>3. Project presentation and publication<br>5. Mentorship	CATCH; RCA; QI processes; Process diagrams; Team Building; Medical error and adverse events; Sentinel events and event reporting; Overview of QI; QI tools; Addressing disparities in QI; Including health equity considerations in QI projects; QI project: using and Interpreting Metrics; Disclosure of adverse events; Human factors engineering; Communication tools; Drills and simulation; Checklists; High reliability organizations; Levels of supervision and progressive increase in authority; Physician well-being; Physician burnout; Professionalism/Fitness for work; Unprofessional behavior; Equity; Freedom from discrimination; Patient-centered and family-centered care; Monitoring of patient care performance indicators	
van Tuijl, 2020 [94]	the Netherlands	Radboud University Medical Center	Physicians, Nurses, Pharmacists, Allied health care, Health care scientists, Health care jurist	Quality and Safety in Patient Care Master program	QI/PS	2 years	1. Didactic lectures (*n*=12)<br>2. Group-based learning and feedback<br>3. Simulation (role play)<br>4. Project-based learning (individual and group projects)<br>5. Coaching<br>6. Peer support	Quality in healthcare; Introduction; Overview; Models; Soft skills; Clinical governance; Patient-centered care; Implementation of QI; Safe care; Efficient care; Scientific research in QI; From project idea to project plan; Personal leadership development; SQUIRE; Transboundary care; Leadership track	2014- ns**

*As described by respective authors.

**Not specified.
